# The evolution of hybrid fitness during speciation

**DOI:** 10.1371/journal.pgen.1008125

**Published:** 2019-05-06

**Authors:** Andrius J. Dagilis, Mark Kirkpatrick, Daniel I. Bolnick

**Affiliations:** 1 Integrative Biology Department, University of Texas at Austin, Austin, Texas, United States of America; 2 Department of Ecology and Evolutionary Biology, University of Connecticut, Mansfield, Connecticut, United States of America; University of Wyoming, UNITED STATES

## Abstract

The evolution of postzygotic reproductive isolation is an important component of speciation. But before isolation is complete there is sometimes a phase of heterosis in which hybrid fitness exceeds that of the two parental species. The genetics and evolution of heterosis and postzygotic isolation have typically been studied in isolation, precluding the development of a unified theory of speciation. Here, we develop a model that incorporates both positive and negative gene interactions, and accounts for the evolution of both heterosis and postzygotic isolation. We parameterize the model with recent data on the fitness effects of 10,000 mutations in yeast, singly and in pairwise epistatic combinations. The model makes novel predictions about the types of interactions that contribute to declining hybrid fitness. We reproduce patterns familiar from earlier models of speciation (e.g. Haldane’s Rule and Darwin’s Corollary) and identify new mechanisms that may underlie these patterns. Our approach provides a general framework for integrating experimental data from gene interaction networks into speciation theory and makes new predictions about the genetic mechanisms of speciation.

## Author summary

As populations diverge and ultimately evolve into distinct species, hybrids between them gradually become inviable and infertile. Before this happens, there can be a brief period in which hybrids are actually more fit than their parents, a condition called heterosis or hybrid vigor. This paper describes a model of how hybrid fitness changes as two populations diverge that can explain both hybrid vigor and hybrid inviability in a single framework. These results show that interactions between alleles within a population can often be more important to hybrid fitness than new interactions first seen in hybrids. Simulations of the model, using state-of-the-art experimental data on gene interactions, show evolutionary trajectories that mirror patterns seen in nature. The model suggests how the process by which populations diverge affects the rate of speciation.

## Introduction

Isolated populations accrue genetic differences. These differences can reduce the fitness of hybrids, constituting a major step the origin of new species [1, 2]. However, important exceptions exist: there are many examples of recently diverged populations whose hybrids are actually more fit than either parent [3–8]. Existing theories of speciation genetics do not provide a unified explanation of the seemingly contradictory observations of heterosis and hybrid incompatibility.

The decline in hybrid fitness is generally attributed to Dobzhansky-Muller incompatibilities, or “DMIs” [9–12]. These are negative epistatic interactions that occur between alleles that are brought together for the first time in hybrids. DMIs have been a major focus of empirical and theoretical studies of speciation genetics [13–15]. Mathematical models of DMIs [16, 17] have generated testable predictions about the evolution of hybrid inviability [16, 18], the asymmetric inviability of reciprocal crosses [19], and sex differences in hybrid fitness [20] (reviewed in [21]). Although these predictions have generally been supported by empirical studies [2], the majority of these models share four limitations. First, they consider only deleterious gene interactions, and so are not able to explain heterosis. Second, many previous speciation models do not account for the mechanism by which mutations become fixed as two species diverge. Substitutions are typically assumed to occur randomly through time, with no explicit consideration of the roles played by selection and drift. How these mutations become fixed could have important consequences for how they affect hybrid fitness. Third, although models of DMI evolution consider the effects of new interactions among genes brought together for the first time in hybrids, they neglect the effects of disrupting beneficial gene interactions that evolved within the parental species. Last, previous models assume interactions between all pairs of genes in hybrids are equally likely, while real gene interaction networks are highly structured [22]. A new wave of models has begun to challenge some of these assumptions individually [23–27]. Here, we develop a more general model that relaxes all four assumptions.

Some recent models have set aside the DMI framework in favor of explaining hybrid fitness using extensions of Fisher’s Geometric Model [27–30]. In such models, mutations have direct effects on phenotypes, and fitness depends on distance to one (or a few) optimal combinations of traits. Appeals of this approach are that hybrid fitness is easily predictable given assumptions about parental fitnesses and it is able to fit empirical data surprisingly well [28]. This framework can explain heterosis as the result of uniparentally inherited elements [27] or low fitness in the parents [28]. Nonetheless, the approach has limitations. Predictions about heterosis are only consistent with speciation under limited assumptions about dominance [28]. More importantly, these models make highly restrictive assumptions about the relationship of genotype to phenotype and the shape of the fitness landscape.

**Fig 1:**
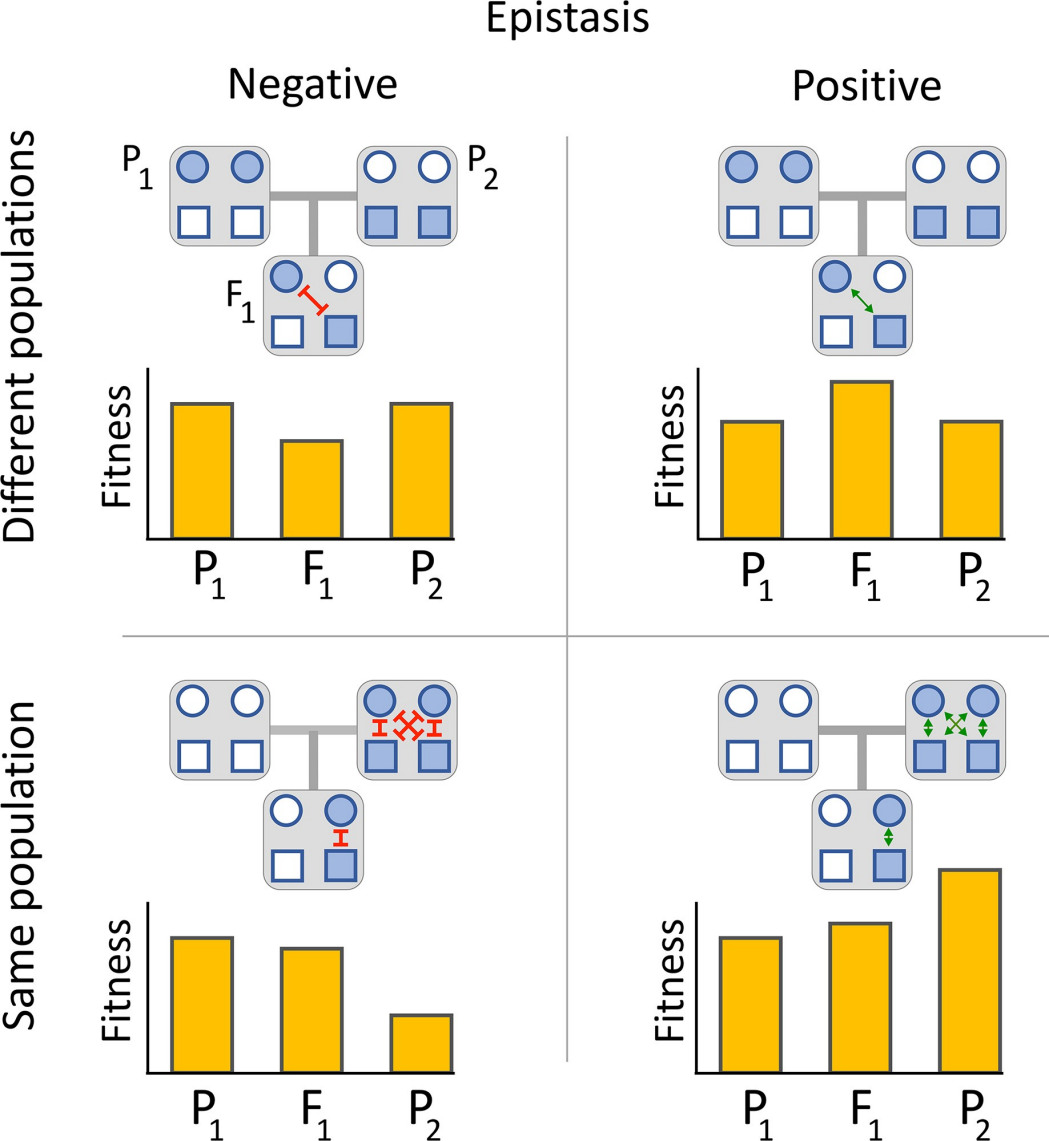
Gene interactions determine parental and hybrid fitness. Parents P_1_ and P_2_ cross to produce a hybrid F_1_ offspring. Each individual carries two loci (circle and square). The ancestral allele is white and derived mutations that have fixed in the two parental species are shaded. If one mutation is fixed in each species (*top panels*), negative epistasis causes decreased hybrid fitness (*top left*), while positive epistasis causes heterosis (*top right*). If two mutations are fixed in one species and none in the other (*bottom panels*), both negative epistasis and positive epistasis cause the hybrid to have intermediate fitness. Depending on the dominance of epistasis, hybrid fitness may be higher or lower than the average of the parents.

Here we develop a framework to model how hybrid fitness evolves as the result of individual and epistatic fitness effects of mutations. Unlike previous DMI models, we allow for positive as well as negative epistatic fitness effects, as shown schematically in Fig 1. We also explicitly model the substitution process. These advances lead to several new conceptual insights. Depending on how mutations become fixed and their spectrum of fitness interactions, hybrid fitness can be higher, lower, or equal to that of the parental species. We find that hybrid fitness is contingent on two classes of epistatic interactions. The first, which has been the focus of previous DMI models, are the new interactions created by hybridization (upper half of Fig 1). The second, which has generally been ignored, are gene interactions present in the two parental species that are disrupted in the hybrids (bottom half of Fig 1).

Under simplified conditions, this framework leads to several analytic results that can explain both heterosis and hybrid incompatibility. We extend the framework with a stochastic model that allows for variation in the effects of mutations and accounts for the probability that a mutation will become fixed in a parental species given its fitness effects. We then parameterize the model with data from recent studies in yeast systems biology. We use the fitness measurements of 10,277 unique single mutants and 20,712,321 double mutants measured in refs. [22, 31, 32] to calculate fixation probabilities and hybrid fitness. The result is the first model for the evolution of postzygotic isolation that is grounded in empirical estimates of fitness effects.

We find that heterosis sometimes occurs and sometimes not. When it does occur, it is always transient and happens early in the speciation process. Heterosis may appear when epistatic interactions within a parental species are deleterious, and their disruption causes F_1_ hybrids to have higher fitness than its parents (Fig 1, bottom left). This case is possible when drift is strong, and mutations with deleterious interactions are fixed in the same population. On the other hand, if epistatic interactions within a parent are beneficial, then F_1_ hybrids can have lower fitness, even in the absence of deleterious DMIs (Fig 1, bottom right). Simulation results show that that the decline of hybrid fitness mainly results from many gene interactions of small effect, which suggests that mapping of genetic incompatibilities will often be difficult. We find that the disruption of co-adapted sets of alleles in a parental species are often more important to determining F_1_ fitness than are novel negative interactions that occur in hybrids. Our study suggests that understanding the structure of epistatic networks may be a crucial prerequisite to predicting hybrid fitness.

## Results

Our model considers two populations that are fixing mutations while diverging in allopatry. Isolation is complete: there is no gene flow between the populations. For simplicity, we assume no single mutation becomes fixed in both populations, and that a negligible number of loci are polymorphic for mutations at any time.

The fitness of an individual, either from a parental population or a hybrid, is a function of the derived alleles (that is, mutations) that it carries. A mutation’s independent and epistatic fitness effects, which can be either positive or negative, are assumed to be multiplicative. Individuals homozygous for mutations at loci *i* and *j* have fitness (1 + *s*_*i*_)(1 + *s*_*j*_)(1 + ε_*ij*_), where *s*_*i*_ and *s*_*j*_ are the independent fitness effects of the mutations, and ε_*ij*_ is the epistatic fitness effect between them when they are both homozygous. An individual that is heterozygous for mutations in set $G_{1}$ and homozygous for mutations in $G_{2}$ has fitness

$$\begin{array}{c} W_{G_{1},G_{2}}=\prod_{i\in G_{1}}\left(1+h_{i}s_{i}\right)\prod_{i\in G_{2}}\left(1+s_{i}\right)\\ \times \prod_{i\in G_{1},j\in G_{2}}\left(1+\alpha_{2}\varepsilon_{ij}\right)\sqrt{\prod_{i,j\in G_{1}}\left(1+\alpha_{1}\varepsilon_{ij}\right)\prod_{i,j\in G_{2}}\left(1+\varepsilon_{ij}\right)}. \end{array}$$ (1)

Here, *h*_*i*_ is the dominance of a mutation at locus *i*. There can be one, two, or four interactions between mutations at a pair of loci, depending on whether the loci are heterozygous or homozygous (S1 Fig). An interaction between two heterozygous derived alleles may be of the same strength as that between two homozygous derived alleles–a form of epistatic dominance. We parameterize epistatic dominance as α_1_ and α_2_ for interactions between two heterozygous loci and between a heterozygous and a homozygous locus, respectively. This approach is similar to the *H*_*0*_, *H*_*1*_ and *H*_*2*_ parameters described by Turelli and Orr [17], but allows us to parameterize our model more easily. The fitness of the common ancestor of the diverging species is defined to be 1, and epistatic interactions in the ancestor are defined to be zero. All fitness effects caused by interactions between a derived mutation at locus *i* and ancestral alleles throughout the genome are accounted for by the independent selection coefficient *s*_*i*_. The square root appears in Eq (1) because each combination of mutations *i* and *j* occurs twice in the products over sets $G_{1}$ and $G_{2}$.

To make our results comparable to empirical studies, we measure the fitness of hybrids relative to the mean fitnesses of the two parental species, and denote relative hybrid fitness as *w*_H_. The expression for *w*_H_ is given in the Methods section.

### Analytical results

We first studied the model analytically by making several simplifying assumptions. Intuitively, we expect mutations that become fixed within a population will tend to have positive interactions with mutations that have previously fixed. If not, then the new mutations would be deleterious and unlikely to fix. We denote the mean of these “within-population epistasis” effects as ${\bar{\varepsilon }}_{w}$, which for simplicity we assume is constant in time. In contrast, the interactions between mutations fixed in different populations will not have experienced this selective sieve, so we expect them to be less positive (and perhaps even negative). We denote the mean of the “between-population epistasis” effects as ${\bar{\varepsilon }}_{b}$, and again assume that it is constant in time. The final simplifying assumptions are that the means and the coefficients of variation of the fitness effects of mutations are much smaller than one, and that independent fitness effects have additive dominance (*h* = 1/2).

Consider the situation when a total of *n* substitutions have fixed in the two populations, a fraction *F* of which fixed in the first population. The relative fitness of hybrids (which is derived in File S1) is then

$$w_{H}≅\exp\left\{{\bar{\varepsilon }}_{b}\alpha_{1}v\;n^{2}-{\bar{\varepsilon }}_{w}(\frac{1}{2}-\alpha_{1})[(\frac{1}{2}-v)n^{2}-\frac{n}{2}]\right\}.$$ (2)

**Fig 2:**
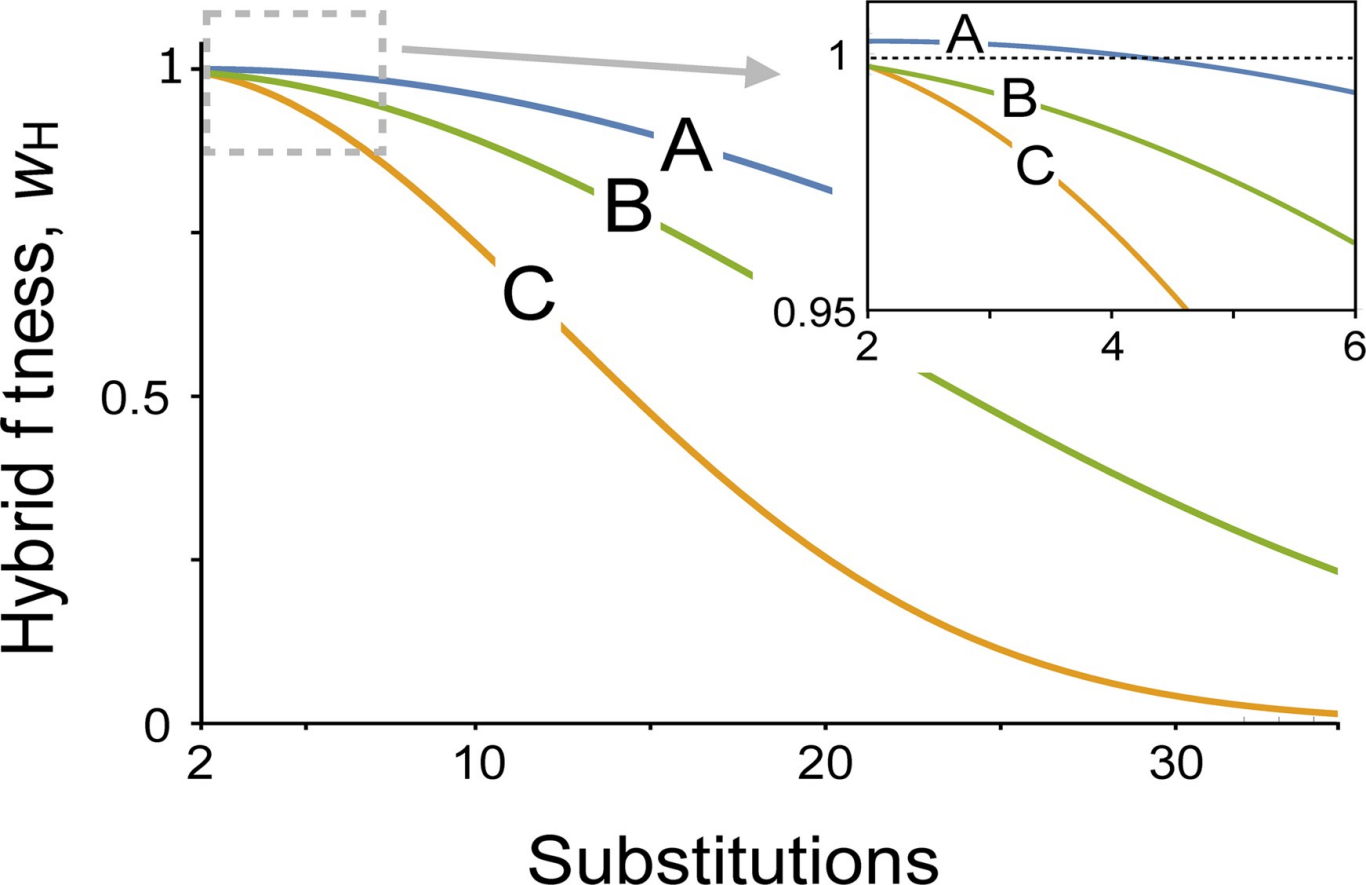
Relative hybrid fitness through time in the analytic model. Results from Eq (2) are shown for three sets of parameters. Heterosis occurs during the first four fixed substitutions with set (A). Parameters values in (A) are ${\bar{\varepsilon }}_{w}$ = 0.02 and ${\bar{\varepsilon }}_{b}$ = 0.01; in (B) are ${\bar{\varepsilon }}_{w}$ = 0.01 and ${\bar{\varepsilon }}_{b}$ = -0.01; and in (C) are ${\bar{\varepsilon }}_{w}$ = 0.05 and ${\bar{\varepsilon }}_{b}$ = -0.01. In all cases the populations are diverging symmetrically (*v* = 1/4) and epistatic dominance is additive (α_1_ = 1/4). See S2 Fig for an exploration of a wider range of parameters.

The parameter *v* = *F*(1 –*F*) measures the symmetry of divergence: *v* = 1/4 when substitutions occur at equal rates in the two populations, and *v* = 0 when substitutions only occur in one population. (See the Supplementary Text for derivations.) Examples of the change of hybrid fitness in time are shown in Fig 2 and S2 Fig.

Eq 2 reveals that hybrid fitness is determined by two factors. First, there are the new gene interactions, represented by the first of the two terms inside the curly brackets (and shown in Fig 1, top panels). These interactions may on average be deleterious (${\bar{\varepsilon }}_{b}<0$), as assumed by previous Dobzhansky-Muller incompatibility (DMI) models, or they may be beneficial (${\bar{\varepsilon }}_{b}>0$). Second, epistatic interactions between mutations that were fixed in each parental population are disrupted in the hybrids, represented by the second product in the brackets (Fig 1, bottom panels). This term has been overlooked in most prior DMI models. The strength of each of these two effects depends on both the dominance of epistasis and the number of mutations fixed in the two populations. When only one population evolves, or when epistatic effects are completely recessive (*v* = 0 or α_1_ = 0), then hybrid fitness is affected solely by the loss of interactions within the parental populations. The epistatic dominance parameter α_2_ does not appear in Eq (2) because all mutations carried by F_1_ hybrids are heterozygous (due to our assumption that polymorphic loci are vanishingly rare). The independent fitness effects of mutations (*s*_*i*_) are also absent because the number of mutations carried by hybrids is equal to the mean number carried by their parents.

Heterosis occurs whenever *w*_H_ is greater than 1. Of particular interest are cases in which heterosis is transient, as seen in empirical studies of some emerging pairs of species [3, 8]. The results from Eq (2) are particularly simple when the two populations evolve at the same rate, and epistatic dominance is additive (*v* = ¼, α_1_ = ¼). Heterosis then occurs whenever both within- and between-population epistasis are on average positive and within-population epistasis is stronger than between-population epistasis (0 < ${\bar{\varepsilon }}_{b}<{\bar{\varepsilon }}_{w}$). Hybrid fitness ultimately declines relative to parents, and heterosis is replaced by postzygotic isolation, after a total of (${\bar{\varepsilon }}_{w}-{\bar{\varepsilon }}_{b}$)^-1/2^ + ${\bar{\varepsilon }}_{w}/({\bar{\varepsilon }}_{w}-{\bar{\varepsilon }}_{b})$ substitutions have occurred.

The potential for heterosis thus depends on the relative effects of within-population and between-population epistasis. A mutation is more likely to fix in one population if it has positive interactions with mutations that are already fixed in that same population, while its potential interactions with mutations fixed in another population are irrelevant to its fixation probability. Between-population effects will therefore generally represent a random draw from the distribution of mutant effects. The within- and between-population effects are in turn expected to be quite different. The interactions between mutations fixed within a population can be influenced by the strength of drift: mutations with negative interactions are more likely to fix when drift is strong, which can cause ${\bar{\varepsilon }}_{w}$ to be negative. Hybrids are rescued from these negative effects, and heterosis can result. Finally, if mutations that fix in one population happen to interact badly with those fixed in the other (so ${\bar{\varepsilon }}_{b}$ is large and negative), then no heterosis will occur, and hybrid fitness can decline rapidly with the fixation of only a small number of mutations.

### Classic patterns in speciation

In a classic model of speciation caused by Dobzhansky-Muller incompatibilities, the decline in hybrid fitness accelerates, or “snowballs”, because the number of possible DMIs grows quadratically with the number of mutations fixed in the diverging populations [33]. This prediction has not been strongly supported by empirical studies, however, which has led to the suggestion that the strength of interactions changes over the course of speciation [34, 35].

Our model suggests a different explanation for the missing snowball. When the strengths of within- and between-population epistasis are equal (${\bar{\varepsilon }}_{b}={\bar{\varepsilon }}_{w}$) and the populations evolve at the same rate (*v* = ¼), hybrid fitness changes in a linear manner as mutations are successively fixed, rather than accelerating. The reason for this discrepancy with the snowball model is quite simple. While the number of new gene interactions in hybrids grows combinatorically, so does the number of beneficial interactions that have evolved in the parental species and that are disrupted in the hybrids. Even when both populations are evolving at equal rates, the number of between-population interactions is only greater than the number of within-population interactions by a small linear factor (*n*/2). Hybrid fitness is therefore determined by this small number of between population interactions. These conditions occur when epistasis is very weak or drift is very strong. In that case, within-population epistasis will not be much affected by selection and will strongly resemble epistasis between populations. Earlier conclusions about the pattern of decline in hybrid fitness failed to separate out the effect of within-population interactions and therefore need reevaluation (although see [36] for a model that only includes within population interactions).

Haldane’s Rule is the observation that when only one sex of hybrids suffers low fitness, it is the heterogametic sex [20, 37, 38]. This pattern is frequently attributed to dominance [39], but a large body of theoretical work has identified recessive interactions between sex chromosomes and autosomes, and faster evolution of the X chromosome, as important drivers (see [40] for a recent review). To learn how this pattern might emerge from our model, we extended it to include sex-linked loci (Section 2d of Supplementary Text). Hybrid female fitness now depends on interactions between mutations that fixed on X chromosomes evolving independently in the two populations, while hybrid male fitness depends on interactions between mutations fixed on the X in one population and mutations fixed on the Y in the other. The relevant comparison for Haldane’s Rule is then to ask whether a diverged X would have more deleterious interactions with an X or Y from a different species (considering only mutations fixed on non-recombining regions of the X and Y). If X mutations tend to compensate for those fixed on the Y (which is highly susceptible to drift), Haldane’s Rule may result due to largely negative epistasis between a Y and an X from different populations. In an important departure from previous theory [17], our model shows that Haldane’s Rule can result if epistasis between diverged Xs is positive even when no mutations are fixed on the Y. This result makes the testable prediction that Haldane’s Rule in XO systems could be explained by positive epistasis between diverged Xs [20]. Our model thus compliments prior explanations for Haldane’s Rule that depend on dominance.

Darwin’s Corollary is the observation that hybrid fitness sometimes depends on the direction of the cross [19, 41, 42]. It is thought this asymmetry results from genetic interactions between one set of mutations on autosomes and another set on the sex chromosomes and/or mitochondria [41, 42]. Extending our model further to include mutations that fix on mitochondria (Supplementary Text, section 2f), we find that the pattern of Darwin’s Corollary emerges whenever one population evolves faster than the other, and is especially prominent if the X or mitochondria evolve faster than autosomes (consistent with the “faster-X effect” [43, 44]).

### Parameterizing the model with data

We next explored the implications of our model when the fitness effects are fit to data from laboratory populations of yeast. We estimated the selection coefficients that appear in Eq (1) using data on the 10,277 knockout mutations studied by Costanzo *et al*. [22], who measured the independent effects and the epistatic effects between 20,712,321 pairs of mutations. These measurements were taken during the haploid phase of the life cycle, so they provide no information about dominance effects. We therefore simply assumed mutations have no dominance (*h*_*i*_ = ½). Further details about the data are given in Materials and Methods.

As observed previously [31], in these yeast there is a significant positive correlation between a mutation’s independent effect and its interactions with other mutations (S3 Fig). Mutations that are highly deleterious individually also tend have highly negative interactions with other mutations. While the biological cause of this correlation remains unclear, it does have an important implication for speciation: pairs of mutations that have strongly negative interactions, and are therefore more capable of killing hybrids, are unlikely to fix in the first place.

This pattern leads to a novel prediction: interactions between mutations fixed early in divergence may contribute less to reproductive isolation than those fixed later. Mutations that appear early will interact with only a small number of mutations that have previously fixed, and so the fate of these mutations is determined mainly by their independent effects. Only those with positive independent effects are likely to fix, and because of the correlation noted above, these early mutations will also tend to have beneficial interactions in hybrids. Later in divergence, the probability that a mutation becomes fixed is mainly determined by how well it interacts with mutations that were previously fixed in that population rather than its own independent effect. It will therefore not be more likely than random to have beneficial interactions with mutations fixed in the other population. Thus within-population epistasis is expected to become increasingly strong and positive, while between population epistasis will tend to become more negative. The result is that the loss of hybrid fitness accelerates. This acceleration resembles the snowball effect discussed earlier [33], but occurs for a very different reason: changes in the average epistatic effects of mutations over time, rather than changes in their numbers.

### A stochastic model

**Fig 3:**
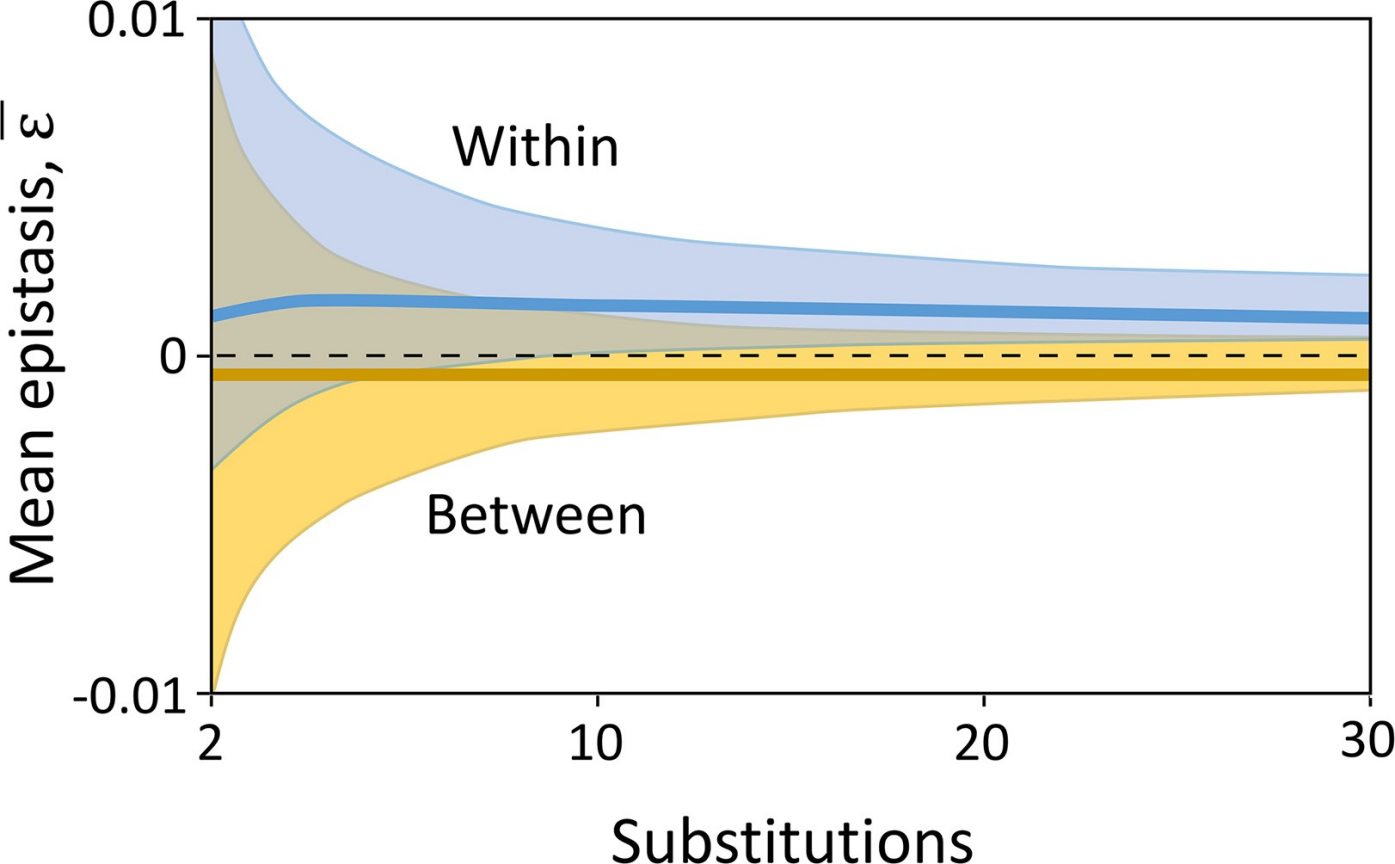
Average epistatic effects of substitutions. Stochastic simulations of the full model show that the average within-population epistasis is positive at all times. It increases during the first few generations of divergence and then stabilizes. The average between-population epistasis is negative and nearly constant, and has a much smaller absolute size than within-population epistasis. The shaded regions represent 95% confidence intervals.

To determine the full evolutionary trajectories of hybrid fitness, we turned to stochastic simulations based on the parameterized model. In the simulations, mutations fix sequentially as the result of selection and drift (details are given in the Methods). As the populations diverge, the fitness effects of mutations that fix are altered by their interactions with mutations that were fixed previously. This change allows the fixation of mutations later in the process of divergence that previously had been highly deleterious (S4 Fig). As a result, each population becomes enriched for co-adapted sets of mutations. In contrast, between-population effects of mutations change little through time because those effects are not tested by selection. These contrasting patterns are shown in Fig 3.

**Fig 4:**
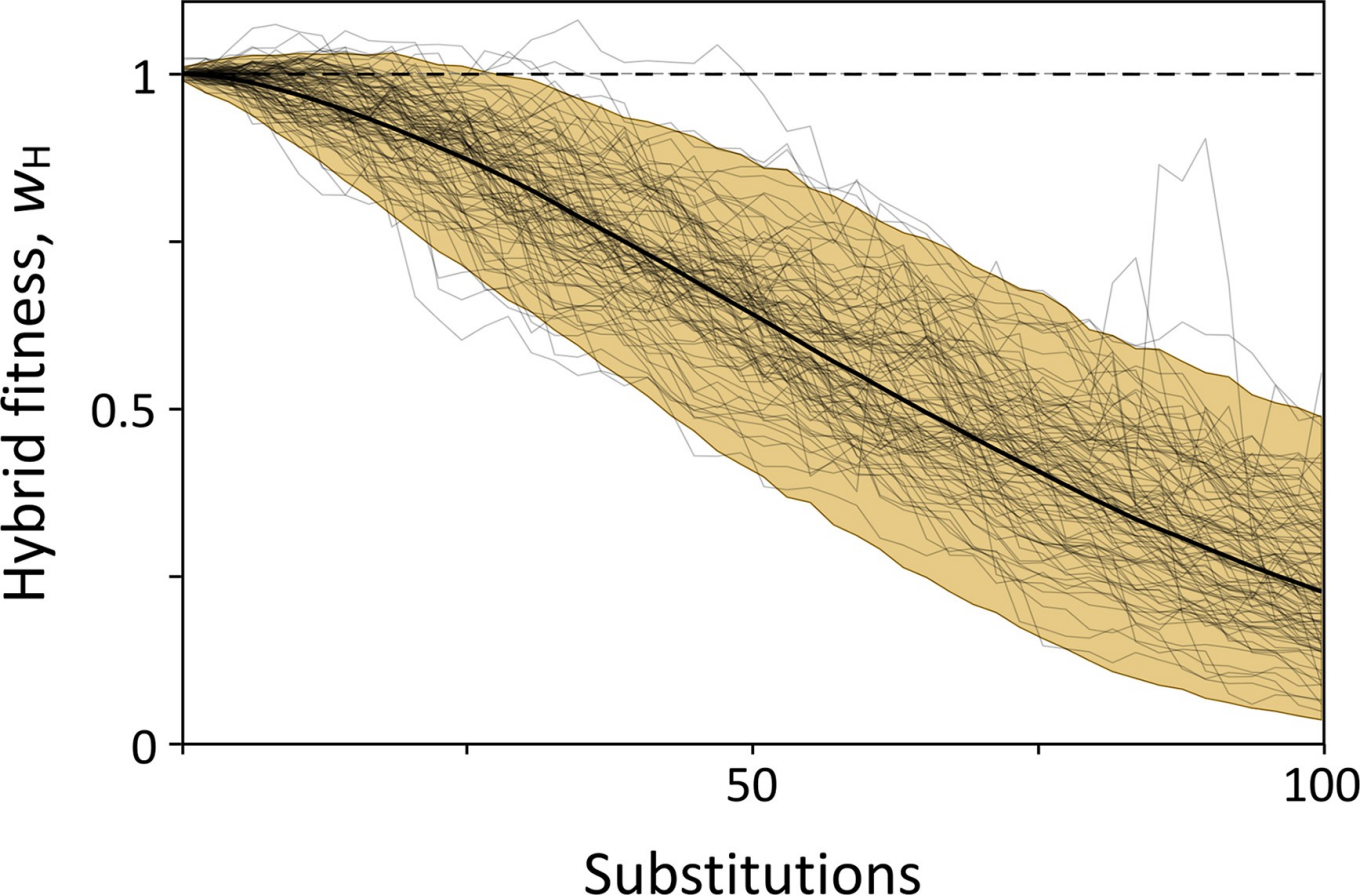
Relative hybrid fitness through time from the stochastic simulations. The solid black curve shows the mean hybrid fitness over 10,000 simulations, the grey lines show a sample of 100 simulations, and the shaded area gives the 95% confidence interval. Heterosis (*w*_H_ > 1) occurs in some simulations early in divergence, but then rapidly disappears as populations continue to diverge.

Fig 4 shows how hybrid fitness evolves when we parameterize our general model with fitness data from yeast. Heterosis is quite common early in the process of speciation: it occurs in fully 10% of the simulations after 10 mutations are fixed in each population. But even in those cases, hybrid fitness inevitably declines. On average, relative hybrid fitness decreases by only 1% with each additional mutation that becomes fixed, and in only 2% of simulations does a single mutation causes a large change (> 10%) at any time during divergence. These results suggest that the genetic mapping of incompatibilities will often be difficult because hybrid fitness is typically determined by many interactions of small effect.

The two dominance parameters for epistatic effects modify our conclusions in several ways. The dominance of epistasis between two heterozygous loci, α_1_, acts to modify the effect of within and between population epistasis to hybrid fitness (S8 Fig). On the other hand, the dominance of epistasis between a heterozygous and a homozygous locus, α_2_, modifies which mutations fix within populations. This greatly affects how hybrid fitness evolves (S9–S12 Figs). Most notably, when α_2_ is very small (epistatic effects are highly recessive), the probability that a mutation becomes fixed is almost entirely based on its independent fitness effect. Mutations that do fix will also have little effect on hybrid fitness (because they are heterozygous in F_1_s). As a result, the average epistatic effects within and between populations are very close to the average effect of all mutations, and to each other (${\bar{\varepsilon }}_{b}\approx {\bar{\varepsilon }}_{w}$). As seen in our analytical results, this leads to linear change in hybrid fitness as the number of substitutions increases.

Lastly, we find that even late in divergence, when few hybrids survive, a large proportion of mutations that have fixed in one population are also beneficial in the genomic background of the other. This fraction declines from roughly 75% when only 2 mutations are fixed in each population to 50% after 50 substitutions have fixed (S5 Fig). Thus, even when hybrids are far less fit than their parents, many of the genes they carry may be able to introgress from one population into the other. Hybridization may thus involve both reduced hybrid fitness overall, but also deterministic introgression for particular alleles.

## Discussion

Although observations of heterosis and postzygotic isolation may appear to be incompatible, the results of this model show they can neatly be explained by the same factors. Hybrid fitness is affected by two kinds of epistatic interactions: those between mutations fixed within a population, and those between mutations fixed in different populations. Each of the two can be beneficial or deleterious. Previous models of speciation have focused on the negative consequences of between-population epistasis, and neglected the consequences of disrupting the interactions between mutations fixed within each parental population.

Our model reveals the importance that within-population epistasis may have in speciation. Data from yeast suggest that interactions between many mutations are positive [22] (although these data are biased for mutations of positive effect), and our results show that these can contribute to heterosis. Positive epistasis also plays a key role in the evolution of reproductive incompatibility because mutations fixed in a parental population are likely to interact positively with each other, and hybrids can miss out on those fitness benefits (Fig 1). Our simulations show that early in divergence, interactions between mutations fixed in a population are typically weak, and disrupting them has little effect on hybrid fitness. But as a population continues to evolve in isolation, it builds up sets of co-adapted alleles, and their positive interactions are disrupted in hybrids. The loss in hybrid fitness caused by this disruption can be an order of magnitude larger than that caused by new deleterious interactions between mutations that first encounter each other in hybrids (Fig 3). In the most extreme cases, the disruption of beneficial interactions within the parent populations could cause hybrid lethality.

The average strength of within-population epistasis (${\bar{\varepsilon }}_{w}$) depends strongly on the roles that selection and drift play in the divergence of the two populations. If epistasis is strong and drift is weak, mutations that fix will tend to have positive interactions, and (as we have seen) their loss can be the major cause of hybrid incompatibility. But when epistatic effects are recessive or very weak and drift is strong, then ${\bar{\varepsilon }}_{w}$ will be similar to the network average. In that case, the disruption of gene interactions that occur in the parental population will play a small role in determining hybrid fitness.

The second type of gene interaction that appears in our model is between-population epistasis. In contrast to within-population epistasis, the average strength of between-population epistasis (${\bar{\varepsilon }}_{b}$) is not determined by adaptation during divergence. Instead, it is expected to represent the effects of random samples from the epistatic interaction network. Our simulations confirm this intuition (Figs 4, S12 and S14).

Another factor that contributes to the evolution of isolation is the relative rates at which the two populations are evolving. Our results suggest that isolation develops more quickly when one population is evolving much more rapidly than the other. In the extreme case when only one population is evolving (and therefore *v* is zero), Eq (2) shows hybrid fitness is determined entirely by the loss of beneficial epistatic effects enjoyed within that population. A follow-up prediction is that backcrossing an F_1_ hybrid with the faster adapting population should restore fitness, while the reciprocal backcross should not.

A final factor that influences the evolution of postzygotic isolation is dominance. Eq (2) shows that interactions between pairs of heterozygous mutations (measured by α_1_) play a direct role in determining hybrid fitness, but interactions between a heterozygous and a homozygous mutation (measured by α_2_) do not. This second parameter does, however, influence which mutations fix, as a rare mutant allele will occur in a genetic background where it is heterozygous but other derived alleles are homozygous. This intuition is confirmed by the simulations (S13 and S15 Figs). To our knowledge, there has been no experimental study of the dominance of epistatic effects, and so these parameters, crucial to determining the trajectory of hybrid fitness, remain unknown. We suspect, however, that deleterious gene interactions are often recessive, because that is the basis of the highly successful theories constructed to explain Haldane’s Rule [11, 40] and the large X effect [17].

### Relations with existing models

Heterosis has previously been explained by overdominance and by rescue from recessive deleterious alleles that have fixed by strong drift [4, 5, 45, 46]. The role of epistasis has received less attention. Studies of natural populations generally do not have sufficient power to detect epistatic effects. Studies in crop plants have found that epistasis may be important to hybrid fitness [47–50], but it is not known whether these findings generalize to natural populations. In nature, heterosis could well result from a combination of the epistatic effects that are the focus of our model and the effects of overdominance and recessive deleterious alleles that have been modeled previously. On the other hand, epistasis seems to be the only hypothesis that can explain why heterosis is frequently seen between closely related species, but not between ones that are more highly diverged. Our model suggests two explanations for this pattern. First, heterosis is transient under simple limiting conditions laid out by Eq 2. Second, in our simulations the variance in epistasis within and between populations is large early in divergence and becomes small as populations continue to diverge (Fig 3). This suggests that heterosis may often be a result of the stochastic sampling of epistatic interactions within and between populations.

Adaptive introgression between hybridizing species has been uncovered in several taxa including *Heliconius* butterflies [51], *Anopheles* mosquitoes [52], and (most famously) in humans [53]. Previous work has shown that DMIs, even of strong effect, may be weak barriers to gene flow across the rest of the genome [54–56], and so even when hybrid fitness is low, introgression may be possible. The genetic consequences of hybridization depend not only on the individual effects that genes have on fitness, but also on how they interact: introgression is aided by positive epistasis and hampered by negative epistasis. In our simulations, fully half of the substitutions in one population can be positively selected in the other even when the populations are nearly completely reproductively isolated (S4 Fig). These results confirm that there is widespread potential for adaptive introgression.

The classic models for the evolution of postzygotic isolation focus on Dobzhansky-Muller incompatibilities (DMIs). A core prediction from these models is the so-called “snowball effect” [16, 18, 57]. This is the pattern in which the loss of hybrid fitness accelerates because the number of new deleterious interactions that occur in hybrids increases quadratically with the number of substitutions that have occurred. The snowball pattern is also predicted by our model under some conditions (Fig 2B and 2C).

There is mixed support for the snowball pattern in natural populations [18, 57], leading to the development of theories to explain the so-called “missing snowball” [24, 34, 35]. Our model is also compatible with the missing snowball. When the populations evolve at the same rate (*v* = ¼) and the average strength of epistasis is equal within and between populations (${\bar{\varepsilon }}_{b}={\bar{\varepsilon }}_{w}$), hybrid fitness declines linearly (rather than accelerating) with the number of mutations that have fixed (*n*). That is because the number of new gene interactions that appear in hybrids is largely offset by the number of co-adapted interactions that occur in the parents that are lost in the hybrids. Thus, even when the total number of incompatibilities increases quadratically, hybrid fitness may change linearly.

Another family of models for the evolution of postzygotic isolation is based on Fisher’s Geometric Model (FGM) [27, 28, 30, 58]. They assume there is a single phenotypic optimum, and that mutations have direct phenotypic effects. In contrast, our model (as well as the models of DMIs) does not make any assumptions about the underlying phenotypes. The price for this generality is that our model has many more parameters. Under some conditions, the two types of models can produce similar patterns of the change in postzygotic with time. There are, however, differences in some predictions. The core difference is the number of incompatibilities. In our model and the DMI models, the number of potential incompatibilities increases at least quadratically as populations diverge. FGM models, on the other hand, predict only a linear increase in the total number of incompatibilities. In practice it is difficult to determine the number of incompatibilities, making this difference hard to test. Our results suggest another contrast between the models: we expect more rapid speciation when one population is evolving much faster than the other, while FGM models make no such prediction.

### Caveats and future extensions

Our model assumes the two populations diverge in complete isolation, and its conclusions might change substantially if there is gene flow between them. If gene flow causes mutations to be tested in the genetic backgrounds of both populations, they may have more positive effects when brought together in hybrids than they do in the absence of gene flow. Conversely, epistatic effects within populations may be less beneficial because a mutation’s survival is affected by its interactions with substitutions in the alternate population. Both effects will tend to cause hybrid fitness to be greater in the presence of gene flow than in its absence, even when the number of mutations that differ between the populations is controlled for.

Our model does not consider interactions between alleles at three or more loci. Recent work on yeast has shown that these higher order interactions may be common, but weaker than pairwise interactions [59]. As populations diverge, the number of higher order interactions increases even more rapidly than the number of pairwise interactions. Consequently, higher order interactions could be the main determinant of hybrid fitness breakdown. Future empirical and theoretical investigation should consider these higher order effects.

We did not study the fitness of F_2_, backcrosses, or later generations of hybrids. It is plausible that later generations may experience both effects of further hybrid breakdown and of transgressive segregation [60–62]. Fitness in later generations depends strongly on the linkage relationships between interacting genes. (For example, if there are sets of interacting alleles that are tightly linked, their contribution to hybrid fitness will break down very slowly as recombination breaks them apart.) Thus, extending this framework to later generations of hybrids requires reformulating the model to account for recombination.

We parameterized the model with data from mutations in yeast that may not be representative. Their fitness effects were measured in the lab, and based on only one fitness component (growth rate). Measurements were made in a single benign environment, so they neglect the potential effects of environmental heterogeneity that may be important to speciation in yeast [63, 64]. Most importantly, our model is of a diploid organism, but the yeast were measured in their haploid state. The dominance of these mutations is unknown, and the distribution of their fitness effects as haploids may not reflect those in diploids. The actual proportion of positive epistasis is difficult to measure and is likely to be different in different species and for different measures of epistasis [65]. Speciation studies in yeast have shown a variety of patterns for both the tempo and cause of speciation in laboratory settings, suggesting the particular rate of speciation observed in our simulations is not universal [4, 64, 66–73]. But while these caveats may challenge the quantitative results, the qualitative results should be robust if the distributions of epistatic effects in nature are similar to those in the data we used.

Here, we have shown that a model of speciation can be parameterized using data from a real biological system. The resulting model can recapitulate basic patterns observed in speciation, including both hybrid incompatibility and heterosis. The model also provides new insights into the genetic basis of postzygotic isolation. Refinements of this modeling framework will be possible when additional data about gene interactions become available, leading to further understanding of the kinetics of speciation.

## Materials and methods

### Data and simulations

To parameterize a stochastic model of evolutionary divergence and speciation, we used the fitnesses in haploids for all 10,858 single knockouts and all 20,712,321 double knockouts in each strain reported by Costanzo *et al*. [22]. The fitness proxy is the colony growth rate of the knockouts. See Costanzo *et al*. supplemental materials for details of fitness measurements and experimental design [22, 32]. Data on the distribution of single-mutation and pairwise epistatic fitness effects in yeast were obtained from the online repository at http://www.thecellmap.org/costanzo2016/.

Our goal in using these data was to estimate the complete distribution of epistatic effects, including weak and absent effects. These weak effects, which were disregarded in the paper reporting the data, may nonetheless be evolutionarily important. To obtain this distribution, we downloaded and concatenated all data files containing fitness measurements including nonessential-by-nonessential genes, nonessential-by-essential, essential-by-essential, and the DAmP experiments. To avoid biasing the dataset against small selection coefficients, we used all measured interactions regardless of their *p*-value.

We write the haploid fitnesses of a single knockout at locus *i* and a double knockout at loci *i* and *j* (respectively) as:

$$\begin{array}{c} W_{i}=\left(1+s_{i}\right),\\ W_{i,j}=\left(1+s_{i}\right)\left(1+s_{j}\right)(1+\varepsilon_{ij}) \end{array}.$$ (3)

Using these relations, we fit the independent selection coefficients and the pairwise epistatic selection coefficients to the yeast dataset. Since some measurements did not report either double knockout fitness or the single knockout fitness of one of the mutations, we were left with 18,743,950 interactions.

To determine which mutation will be the next to become fixed, we calculated the fitness of mutations at all loci in the genome not already fixed for mutations. The fitness of a mutation depends on both its individual effect and its interactions with mutations already fixed (as described by Eq 1). Thus, the fitness of a given mutation changes in time as the genetic background of its population evolves with the fixation of mutations at other loci. The fitnesses of all new mutations were used to calculate their probability of fixation using a standard diffusion approximation, and assuming an effective population size of 10^6^. Finally, a mutation was randomly chosen for fixation with probability proportional to the chance that it would become fixed. This process was iterated until each population was fixed for 50 mutations. The entire process was replicated in 10,000 stochastic simulations.

Analyses and simulations were performed in R, and figures were generated using ggplot2 and cowplot packages [74–76]. The scripts used to generate the simulation data and perform the following analyses are available at https://github.com/adagilis/SpeciationSimulation2018.

### Analytical results

We consider two populations that are diverging in isolation and independently become fixed for different sets of mutations. To assess hybrid fitness as the populations, diverge, we assume that the number of loci at which mutations can fix is so large that there is a negligible chance the same mutation will become fixed independently in both populations. Denote as A and B the sets of loci at which mutations have fixed in populations *A* and *B*, respectively, and assume that neither population is polymorphic for derived mutations. We measure hybrid fitness relative to the mean of the two parental species:

$$w_{H}=\frac{W_{(A\cup B),\emptyset }}{\frac{1}{2}(W_{\emptyset ,A}+W_{\emptyset ,B})},$$ (4)

where $W_{X,Y}$ is the absolute fitness of an individual that is heterozygous for mutations at loci in set X and homozygous for loci in set Y, ∪ represents the union operator, and ∅ is the empty set. The numerator is the absolute fitness of an F_1_ hybrid, which is heterozygous for mutations at loci in the set (A∪B), and homozygous for no mutations. The denominator represents the mean of the absolute fitnesses of the parental species. Individuals from population *A* are heterozygous for mutations at no loci, but homozygous for mutations at loci in set A, and likewise for individuals from population *B*.

### Divergence in allopatry

To model allopatric population divergence, we calculated the probability of fixation for each mutation in the yeast genome using Kimura’s diffusion approximation [77]. This probability depends on the mutation’s fitness in heterozygous and homozygous states. Since the strength of selection on each mutation will depend on the genetic background in which it occurs, we use the effective selection coefficient and the effective dominance of each mutation. Let $\sigma_{i|A}$ and $\omega_{i|A}$ be the effective selection coefficient and dominance for a mutation at locus *i*, which accounts for the mutation’s independent fitness effect and its epistatic interactions with mutations at loci in the set A that have already fixed. Then:

$$\begin{array}{c} \sigma_{i|A}=(1+s_{i})\prod_{j\in A}\left(1+\varepsilon_{ij}\right)-1,\\ \omega_{i|A}=\frac{[(1+{h_{i}s}_{i})\prod_{j\in A}\left(1+{\alpha_{2}\varepsilon }_{ij}\right)]-1}{\sigma_{i|A}}. \end{array}$$ (5)

We assume that polymorphism is sufficiently rare that a population can be assumed fixed at all other loci when considering the fixation probability of a new mutation. (An interesting extension for future work would relax the assumption of no polymorphism [78].) When no other mutations have yet been fixed, $\sigma_{i|A}$ is equal to *s*_*i*_ and $\omega_{i|A}$ = *h*_*i*._

## Supporting information

S1 Text Supplementary Text. This file contains details about the simulation results, additional simulations under varying assumptions about epistasis and analytical derivations for the model.
(DOCX)

S1 Fig Epistatic dominance parameters. Schematic of the 3 possible strengths of epistatic interactions when (from left to right) both loci are heterozygous for derived (shaded) allele, one is heterozygous and one is homozygous, and both are homozygous. The red lines show the epistatic interactions. These values correspond to Turelli and Orr’s notation of H_0_, H_1_, H_2_ from left to right, respectively.
(TIF)

S2 FigAnalytical solutions for relative hybrid fitness.The effect of varying the strength of epistasis, asymmetry in divergence and dominance of epistasis to hybrid fitness. All heat maps are numerical evaluations of hybrid fitness given by Eq (2) of the main text, with default parameter values ${\bar{\varepsilon }}_{b}$ = -0.005, ${\bar{\varepsilon }}_{w}$ = 0.01, v = 1/4, α1 = 1/4. Varying ${\bar{\varepsilon }}_{b}$ (top left) causes hybrid fitness to decline more rapidly when between population epistasis becomes more deleterious, and heterotic when they are positive. The inverse pattern is observed for ${\bar{\varepsilon }}_{w}$ (top right). Increasing asymmetry (lower *v* values, bottom left) decreases hybrid fitness more rapidly, while increasing epistatic dominance (bottom right) retains within population epistasis causing heterosis (since $\left|{\bar{\varepsilon }}_{b}|<|{\bar{\varepsilon }}_{b}\right|$), while lower dominance causes more rapid speciation.(TIF)

S3 Fig Average epistatic effects of mutations in the yeast data set. In yeast knockouts, the mean epistatic effect of mutations correlates with the mutations’ direct fitness effects. Each point is one of the mutations in the yeast epistatic interaction data-set. The X-axis is the direct selection coefficient observed in lab experiments, while the Y-axis is the average of all epistatic interactions involving that mutation with all other genes. The red line is the linear fit to the data (adjusted R-squared of 0.103, p<10^−16^).
(TIF)

S4 Fig Fixation rates of mutations in simulations. Observed simulation fixation rates for the 10588 mutations in the yeast epistatic network dataset. Points in red are mutations with on average positive epistasis, while points in blue are epistatically neutral or deleterious. Mutations with even highly negative direct selection coefficients are fixed frequently when their epistatic effects are largely positive.
(TIF)

S5 Fig The fraction of substitutions that may adaptively introgress. The fraction of substitutions in one population that are positively selected in the other population over the course of the simulation. Blue line is the mean of 10000 simulations, grey area is the 95% confidence interval. Nearly all substitutions fixed early are beneficial in the ancestral background and so will fix in both populations if gene flow allows the derived allele to cross between populations. As divergence continues, fewer and fewer of the substitutions in one population are positively selected in the genomic background of the other population. The proportion of positively selected mutations remains high even when hybrid fitness is expected to be very low (<0.2, Fig 4).
(TIF)

S6 Fig The distribution of measures of relative contributions of interactions to hybrid fitness early and late in divergence. The distribution of γ_2_ (A) and γ_100_ (B) values, representing the potential of each individual interaction to contribute to speciation after a total of 2 and 100 mutations have been fixed in two independently evolving populations. Since γ is a cross product of the frequency an interaction is seen in a hybrid and its fitness effects in that hybrid, positive values represent potential for heterosis, while negative ones represent reproductive isolation. The vast majority of interactions are unlikely to contribute to either process.
(TIF)

S7 Fig The importance of interactions early vs late in divergence. The relationship between γ_2_ and γ_100_ for all interactions. Most interactions have no effect towards hybrid speciation either early or late in the process of speciation. The interactions that are most important early in the process (large absolute γ_2_) do not remain important as the populations continue to diverge. This suggest that different interactions may be contributing to hybrid fitness at different times during the process of speciation.
(TIF)

S8 Fig Summary of epistasis in the yeast data assuming additive epistasis. Summary of network using additive epistasis measures. A) The distribution of epistatic effects. B) The proportion of positive epistatic interactions as a function of the cutoff of the absolute value of the interaction. Additive measures of epistasis lead to far fewer positive interactions of large effect, reducing the proportion of positive interactions in the network to negligible amounts if strong cutoffs are considered. C) The highly linear relationship between additive epistatic effect and direct fitness effects of mutations. D) The relationship in C is derived from the strong pattern of deleterious mutations having strongly deleterious interactions, while positively selected interactions tend to occur between positively selected mutations. E) Since positively selected mutations largely have positive interactions, the interactions likely to occur early in hybrids are on average positive.
(TIF)

S9 Fig Simulations of the yeast data set assuming additive epistasis. The simulation results for the additive epistatic model. (A) Under the additive model, only interactions of positive direct fitness effect tend to be fixed. (B) Since positively selected mutations share largely positive epistatic interactions, within and between population interactions slowly converge to the same values. (C) Hybrid fitness asymptotes under the additive model, since novel interactions first seen in hybrids are of roughly the same positive strength as the co-adapted interactions seen within populations (as seen in B).
(TIF)

S10 Fig Epistasis in the yeast data set using a modified version of multiplicative epistasis. Results using the measures of epistasis presented in Costanzo et al. (2016). A) The distribution of epistatic effects. B) The proportion of positive epistatic interactions as a function of the cutoff of the absolute value of the interaction. C) The relationship between additive epistatic effect and direct fitness effects of mutations. The two are weakly but significantly correlated (r^2^ = ,p<10). D)The largest epistatic effects are seen between mutations of most extreme effects (both positive and negative). E) Interactions likely to occur early in speciation are of weaker and more positive effect than interactions that are unlikely to occur early on.
(TIF)

S11 Fig Simulation results using a modified model of multiplicative epistasis. The simulation results for the Costanzo epistatic model. Results are qualitatively similar to the results presented in the main paper, with several exceptions. (A) The modified epistatic model results in mutations with extremely strong direct fitness effects rarely being fixed. Mutations with positive or negative epistatic effect are colored blue and red, respectively. (B) Epistatic effects between populations (red) converge to the average epistatic effect between populations as substitutions are fixed, while interactions within each population (blue) quickly become positive in the majority of the simulations. The curves capture the range of 95% of the simulations. (C) Hybrid fitness decreases more slowly than in the multiplicative case, but much faster than in the additive model. Dashed line represents simulation mean; faint grey lines give representative simulation contours.
(TIF)

S12 Fig Relative hybrid fitness varies with dominance of epistasis in hybrids. Relative hybrid fitness when assumptions about dominance of epistasis are modified. We consider our simulations in which α_2_ = 0.5. Since hybrids will only experience epistasis of strength α_1_ε, we reanalyze their fitness assuming epistasis seen in hybrids is recessive (blue, α_1_ = 0), additive (yellow, α_1_ = 1/2 α_2_) and dominant (red, α_1_ = α_2_). Solid lines represent means of simulations, shaded areas are 95% confidence intervals. As suggested by our model, highly dominant interactions lead to slower decline in fitness. Simulations demonstrate that a secondary effect is increased variance in observed hybrid fitness.
(TIF)

S13 Fig Average epistasis in simulations assuming recessive epistatic interactions. Average epistatic effects within (purple) and between (tan) populations. While variance of within population effects is lower than that of between populations, the two rapidly converge to the same slightly negative value. Shaded areas represent the 95% confidence interval of 2000 simulations, solid lines are means.
(TIF)

S14 Fig Relative hybrid fitness in simulations assuming recessive epistatic interactions. Relative hybrid fitness in simulations when epistatic effects are nearly completely recessive as mutations are fixed (α_2_ = 0.01). Hybrid fitness depends very little on whether epistasis seen in hybrids is recessive (blue, α_1_ = 0), additive (yellow, α_1_ = 1/2 α_2_), or dominant (red, α_1_ = α_2_). Hybrid fitness therefore increases linearly with respect to fixed substitutions, proportional to the average epistatic effect.
(TIF)

S15 Fig Average epistatic effect in simulations assuming dominant epistatic interactions. The average epistatic effect found among mutations fixed in the same population (purple) or in different populations (tan), when epistatic effects are largely dominant. Few deleterious epistatic effects can be fixed within the same population, and so epistasis within rises rapidly and continues to increase as populations diverge. Between population epistasis behaves the same as in simulations where epistasis additively dominant. Shaded areas represent the 95% confidence interval of 2000 simulations, solid lines are means.
(TIF)

S16 Fig Relative hybrid fitness in simulations assuming dominant epistasis. Relative hybrid fitness in simulations when epistatic effects are nearly completely dominant as they are fixed (α_2_ = 0.95). Because within population epistasis is large, hybrid fitness depends heavily on whether epistasis seen in hybrids is recessive (blue, α_1_ = 0), additive (yellow, α_1_ = 1/2 α_2_), or dominant (red, α_1_ = α_2_). When effects are recessive, hybrids lose out on co-adapted blocks and rapidly lose fitness, while interactions that display nearly the same strength as seen in parents (red) lead to extreme heterosis due to combinations of positive epistatic effects from both parents.
(TIF)
