# Supplementary material for: The evolution of hybrid fitness during speciation
Source: PLoS Genet. 2019 May 6;15(5):e1008125. doi: 10.1371/journal.pgen.1008125 (PMC6502311; doi:10.1371/journal.pgen.1008125)
Supplement: S1 Text — This file contains details about the simulation results, additional simulations under varying assumptions about epistasis and analytical derivations for the model. (DOCX) [file pgen.1008125.s001.docx]

**S1 Text: Supplementary Text for “The evolution of hybrid fitness during speciation”**

This document is comprised of two major sections. The first gives supporting results based on the stochastic simulations. The second gives supporting analytic results that pertain to several key phenomena in speciation. These include heterosis, Haldane’s Rule [1-3], and Darwin’s Corollary [4-6].

1. Simulation Results

1.a *Early vs. Late Speciation*

We characterized the potential that a pair of loci have to contribute to speciation very soon after the populations begin to diverge. The expected effect on hybrid fitness caused by an interaction between mutations at loci *i* and *j* is given by the probabilities that these mutations fix times their epistatic effect. Denoting this expected effect as γ_2_(*i*, *j*), then:

γ_2_(*i*, *j*) = 2 *f_i_ f_j_* ε*_ij_* , (S1)

where the fixation probabilities *f_i_* and *f_j_* are evaluated using *s_i_* and *s_j_*, respectively, for the effective selection coefficients. Large absolute values of γ_2_ suggest that an interaction is both likely to occur early in hybrids and have a large fitness impact. Positive values contribute to hybrid vigor (heterosis), and negative values contribute to reproductive isolation.

We investigated the relative importance of certain mutations and interactions in the process of speciation in the long term. For each pair of loci *i* and *j*, we calculated *S_ij_*(*n*), defined as the frequency with which fitness interactions occurred between mutations at those loci within the same population. This quantity is equal to the number of fixations at both loci *i* and *j* in the same population divided by the total number of simulations. We likewise calculated *D_ij_*(*n*), the frequency with which new epistatic interactions occur between loci *i* and *j* in hybrids, given that a total of *n* mutations have fixed ( = number of times mutations at loci *i* and *j* fix in different populations divided by the number of simulations). Positive interactions that are frequently observed within the same population have great potential to be broken up in a hybrid, thus contributing to reproductive isolation because hybrids lack beneficial interactions present in one or both parental species. On the other hand, negative interactions that are only seen in hybrids have potential to cause novel incompatibilities.

We define γ*_n_*(*i*, *j*) describing the potential for speciation by an interaction after a total of *n* interactions are fixed, half of which are fixed in each population:

$\gamma_{n}\left( i,j \right)=\varepsilon_{ij}\left( \frac{{4D}_{ij}\left( n \right)}{n^{2}}-\frac{S{}_{ij}\left( n \right)}{n(n/2-1)} \right)$. (S2)


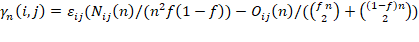


The terms *D_ij_*(.) and *S_ij_*(.) account for the total number of old and new interactions possible. Equations (S1) and (S2) are equivalent for *n*=2. In the main text, we focus on *n* = 100.

Hybrid fitness is partly determined by epistatic interactions between mutations that have been fixed in the two parental populations. During the earliest stages of divergence, the potential that given pair of loci interact to reduce hybrid fitness is quantified by our statistic γ_2_. It shows that the vast majority of potential interactions are very unlikely to contribute to speciation. This is because either the interaction between the pair of loci is very weak, or mutations at these loci are deleterious within the parental populations and so very unlikely to fix.

Early after the two populations are isolated, the probability that a mutation becomes fixed within either population is largely determined by its direct selection coefficient, *s_i_*. Later in divergence, that same mutation also can interact epistatically with previously-fixed mutations. So as divergence proceeds, the identity and number of substitutions increasingly changes the probability that any particular new mutation will fix. We found, however, that the direct effect still has a large impact on fixation probabilities after 50 mutations are fixed in each population. The correlation between the initial fixation probability (that is, with no other mutations fixed) and the probability after 100 mutations fix is *r* = 0.77 (*p* < 10^-12^).

Because of this changing fitness landscape as divergence proceeds, we found that the interactions likely to play a role early in speciation are unlikely to do so in the long run. And, genes that are likely to contribute to speciation later in divergence are unlikely to evolve early on. The potential that mutations at a pair of loci will contribute to isolation late in the speciation process is quantified by our γ_100_ statistic. It is negatively correlated with γ_2_ (*r* = -0.24). Many interactions that are important early in divergence have γ_100_ values close to 0.

1.b *Alternative fitness models*

The results described in our main text are based on the multiplicative model for epistasis given by equation (1). Multiplicative fitness models necessarily bind the minimum fitness to 0, while additive models can result in negative fitness when many interactions of weak effect contribute to fitness. Mixed models may have some effects (such as direct fitness effects) be multiplicative, while epistatic effects are additive. We focused our main text on multiplicative models because these seem biologically most plausible. However, to evaluate the consequences of changing this assumption, in this section we consider two alternative fitness schemes.

Fitness can be parameterized in terms of additive rather than multiplicative selection coefficients. Doing so changes some of our quantitative conclusions, but has little effect on the qualitative conclusions. Equation (S1) can be replaced with:

$W_{\mathbb{G}_{1},\mathbb{G}_{2}}=1+\sum_{i\in\mathbb{G}_{1}} h_{i}s_{i}+\sum_{i\in\mathbb{G}_{2}} s_{i}+\frac{\alpha_{1}}{2}\sum_{i,j\in\mathbb{G}_{1}} \varepsilon_{ij}+\frac{\alpha_{2}}{2}\sum_{i,j\in\mathbb{G}_{2}} \varepsilon_{ij}+\sum_{i\in\mathbb{G}_{1},j\in\mathbb{G}_{2}} \varepsilon_{ij}$. (S3)

We estimate the direct and epistatic selection coefficients by

*W_i,j_* = 1 + *s*_i_ + *s_j_* + ε*_ij_* . (S4)

We again calculated the fixation probabilities *f_i_* and the γ_2_ for all interactions. We assumed that dominance was completely additive (*α*_1_=1/4, *α*_2_=1/2 _,_*h*=1/2). We ran 10,000 simulations of 50 substitutions fixed in each population.

The additive model of epistasis shows a strong linear relationship of the direct fitness of mutations vs. their epistatic effects (Fig S4C). Mutations with positive direct benefits have positive genetic interactions, while even highly positive mutations are on average deleteriously epistatic under the multiplicative model. As a result we find that hybrid fitness seems to asymptote, and potentially even increase as more mutations are fixed (Fig S4C). This pattern is driven by the relatively equal strengths of interactions between populations that are first seen in hybrids, and interactions within populations which are diluted in those hybrids (Fig S4B).

A second alternative way to parameterize fitness comes from the statistical model used by Costanzo *et al.* [7]. Their fitness model is a mixture of our additive and our multiplicative models. This model has the benefit of minimizing the magnitude of epistatic effects when direct fitness coefficients are highly deleterious. For example, if two mutations reduce fitness by 80%, the expected double mutant fitness is 0.04 of the reference strain. Minor deviations due to experimental noise can therefore modify the magnitude of the epistatic effect greatly, while the Costanzo et al method limits the possible strength of the epistatic interaction. The direct and epistatic selection coefficients are defined as:

*W_i,j_* = (1 + *s_i_*)(1 + *s_j_*) + ε*_ij_* . (S5)

In terms of our notation, their assumptions correspond to the fitness function:

$$W_{\mathbb{G}_{1},\mathbb{G}_{2}}=\prod_{i\in\mathbb{G}_{1}} (1+{h_{i}s}_{i})\prod_{i\in\mathbb{G}_{2}} (1+s_{i})+$$

$\alpha_{1}\sum_{i,j\in\mathbb{G}_{1}} \varepsilon_{ij}+\alpha_{2}\sum_{i,j\in\mathbb{G}_{1}} \varepsilon_{ij}+\frac{1}{2}\sum_{i\in\mathbb{G}_{1},j\in\mathbb{G}_{2}} \varepsilon_{ij}.$ (S6)

Note that the epistatic terms are additive, while direct fitness effects are multiplicative.

We re-ran many of our analyses using these alternative epistasis models, assuming completely additive epistasis (*α*_1_=1/4, *α*_2_=1/2 _,_*h*=1/2). Results from simulations based on this parameterization are qualitatively similar to those from the multiplicative model (Figs S5 and S6). There are minor discrepancies, similar to those seen under the additive model.

1.c *Alternative dominance models*

The results in the main text are presented with the assumption that the dominance effects of direct and epistatic fitness are both completely additive. This simplifying assumption can be modified in several ways. Since there are two dominance parameters for epistasis (α_1_ and α_2_; Fig S2), we first consider the consequence of modifying α_1_. An interaction between two derived alleles that are heterozygous may be completely masked by dominant ancestral-ancestral or derived-ancestral interactions. As we show in equation (S9), hybrid fitness only depends on α_1_ directly, as F_1_ hybrids only experience interactions between two heterozygous derived mutations. However, the strength of epistasis between a heterozygous and homozygous derived mutation will be crucial to which mutations are fixed, as that is the background they are likely to occur in while rare. Thus, while α_1_ impacts hybrid fitness directly, α_2_ may be more important to shaping *which* interactions occur within and between populations. We therefore examine three cases of α_2_ and assume that α_1_ = 0, 0.5α_2_, or α_2_ to test the effects of dominance. We first reanalyze our general simulations assuming α_2_= 0.5 (Fig S8). As expected from our analytical model, α_1_ acts as a scaling factor, with speciation proceeding more slowly when interactions are more dominant.

When epistasis is recessive, hybrids will suffer a greater loss of any co-adapted sets of loci. However, recessive epistasis also makes it unlikely that co-adapted blocks of mutations are fixed. To test this intuition, we ran 2000 simulations assuming α_2_=0.01. Populations in these simulations fixed exclusively mutations with positive direct selection coefficients, while epistasis within vs between populations was not significantly different (Fig S13). The result is that hybrids experience a minor increase in fitness relative to parental populations, since the weakly deleterious epistatic interactions in the parents are significantly diluted in the hybrid (Fig S14). Because this steadily increasing hybrid fitness through time is contradicted by empirical fact, recessive epistasis is probably not typical. See section 2b for a discussion of how speciation dynamics play out when epistasis within is equal to epistasis between populations.

When epistatic effects are dominant, hybrids will not lose out on co-adapted blocs of interactions as much as in the standard model, and will experience novel deleterious interactions near their full strength. However, the hybrids will also experience co-adapted interactions from two populations rather than one, giving a large potential for heterosis. We ran simulations assuming that α_2_=0.95. Between-population epistasis is untested by selection, and therefore unaffected, while within population epistasis is strongly optimized due to strong effects in heterozygotes (Fig S15). This leads to very large variance in hybrid fitness (Fig S16), but an overall trend of heterosis in hybrids.

The dominance assumptions used here are rather extreme, but suggest that dominance of epistatic effects is likely to play a strong role in the value of within population epistasis. Recessive epistatic effects will be recessive not only in hybrids, but also in the diverging populations, leading to less chance of epistasis playing a strong role in fitness and speciation. Dominant effects may lead to stronger novel deleterious interactions in hybrids, but also allows beneficial epistasis that evolved within parental populations to be maintained in hybrids. These results highlight the very large role of epistatic-dominance for both within-population adaptation and speciation. Yet, there exists remarkably little data on the topic. Our model suggests that a better understanding of speciation will rely on greater empirical attention to the dominance of epistatic effects.

We performed no tests on the effects of dominance of direct fitness effects. We do not know of a study comparing the relative correlation between dominance in direct vs epistatic effects. Models of both speciation and heterosis often consider dominance in direct effects [8], but more information is needed before the relative contribution of dominance vs epistasis can be evaluated.

2. Analytic Results

This section develops analytic results that pertain to several key features of speciation. We first investigate how hybrid fitness will change as populations diverge. We show that under some simplifying assumptions hybrid fitness changes in a very predictable fashion. We compare the predictions of our model with previous speciation models, and outline the conditions under which heterosis is expected. We then evaluate what conditions are required for our model to yield some of the commonly documented empirical patterns in speciation:

We investigate Haldane’s Rule, the observation that hybrids of the heterogametic sex typically show lower fitness [1-3]. The leading hypothesis for Haldane's Rule is that recessive interactions with alleles on the X chromosome are unmasked in males, where those alleles are hemizygous [9]. Our model cannot address that mechanism because we assume there is no dominance. Nevertheless, we show below that Haldane’s Rule does emerge from our model under some conditions, for previously overlooked reasons.

We then examine the idea that cyto-nuclear interactions may play a disproportionate role in hybrid fitness [10, 11]. This pattern has often been attributed to novel deleterious interactions between the cytoplasm of one parent and the nuclear genome of the other. We find that if cytoplasmic loci diverge rapidly, cyto-nuclear interactions become increasingly dominant factors in determining hybrid fitness.

Finally, we show that uniparentally transmitted elements such as sex chromosomes and cytoplasmic factors can lead to Darwin’s Corollary – the pattern in which hybrids resulting from reciprocal crosses have different fitness. We show that uniparentally inherited genes contribute to hybrid fitness in ways distinct from the rest of the genome. These distinctions lead to Darwin’s Corollary whenever there is asymmetry in the rate at which the two populations evolve.

2.a *General Results*

We begin by simplifying our fitness function. Recall that equation (1) gives the fitness of an individual heterozygous for mutations in set 𝔾_1_ and homozygous for mutations in set 𝔾_2_. When all the ε*_ij_* and *s_i_* are much smaller than 1, then hybrid fitness is well approximated by:

$W_{\mathbb{G}_{1},\mathbb{G}_{2}}\cong\exp\left\{ \sum_{i\in\mathbb{G}_{1}} {h_{i}s}_{i}+\sum_{i\in\mathbb{G}_{2}} s_{i}+\frac{\alpha_{1}}{2}\sum_{i,j\in\mathbb{G}_{1}} \varepsilon_{ij}+\frac{\alpha_{2}}{2}\sum_{i,j\in\mathbb{G}_{2}} \varepsilon_{ij}+\sum_{i\in\mathbb{G}_{1},j\in\mathbb{G}_{2}} \varepsilon_{ij} \right\}$. (S7)

We again assume no overlap between mutations fixed in populations A and B, and that polymorphism is sufficiently rare that a mutation’s fate is determined by its fitness in a genetically uniform background. The fitness of an individual in population A, denoted *W*_A2_, is given by:

$W_{A_{2}}\cong\exp\left\{ \sum_{i\in\mathbb{A}} s_{i}+\frac{1}{2}\sum_{i,j\in\mathbb{A}} \varepsilon_{ij} \right\},$ (S8)

where 𝔸 is the set of loci with mutations that have been fixed in population A since the two populations became isolated. An expression for *W*_B2_, the mean fitness of an individual in population B, is found by replacing 𝔸 by 𝔹 on the right side of (S6).

The fitness of a hybrid relative to the average fitness of individuals from the parental populations is:

$w_{H}=\frac{\bar{W}_{A_{1}B_{1}}}{W_{A_{2}}+W_{B_{2}}}\cong\exp\left\{ \begin{aligned} \left( h-\frac{1}{2} \right)\sum_{i\in\mathbb{A\cup B}} s_{i}+\alpha_{1}\sum_{i\in\mathbb{A}.j\in\mathbb{B}} \varepsilon_{ij}+ \\ \left( \alpha_{1}-\frac{1}{2} \right)\left( \sum_{i,j\in\mathbb{A}} \varepsilon_{ij}+\sum_{i,j\in\mathbb{B}} \varepsilon_{ij} \right) \end{aligned} \right\}.$ (S9)


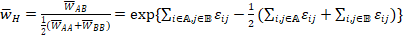


Direct selection effects only play a role if dominance is not fully additive. Recessive mutations lead to hybrids experiencing a loss of the direct fitness effects experienced in parental populations. If *h* = 1/2, all direct effects are canceled out and equation (S9) reduces to:

$w_{H}=\frac{\bar{W}_{A_{1}B_{1}}}{W_{A_{2}}+W_{B_{2}}}\cong\exp\left\{ \alpha{}_{1}\sum_{i\in\mathbb{A}.j\in\mathbb{B}} \varepsilon_{ij}-\left( \frac{1}{2}-\alpha_{1} \right)\left( \sum_{i,j\in\mathbb{A}} \varepsilon_{ij}+\sum_{i,j\in\mathbb{B}} \varepsilon_{ij} \right) \right\}.$ (S10)


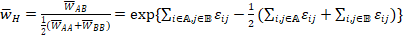


In biological terms, the relative fitness of the hybrid is determined by a balance between two kinds of epistatic fitness effects. One is represented on the right side of (S10) by the first summation in the exponent. These are new epistatic effects created in the hybrid by novel combinations of alleles introduced from the two populations. Such new epistatic effects represent the basis of the Dobzhansky-Muller model.

Equation (S10) reveals that a second type effect of epistatic effect also impacts hybrid fitness. It is represented by the two summations within the parentheses. These are epistatic effects between mutations fixed within a source populations that are lost in the hybrid. This second type of effect has not been accounted for in previous models for the evolution of hybrid incompatibilities [12-15]. A single model has looked specifically at such interactions, but ignored novel interactions in hybrids [16].

We can ask how relative hybrid fitness changes with the total number of mutations fixed in either population. We generalize the simulation model to allow for different numbers of mutations to be fixed in the two populations. Write the numbers of substitutions in the two populations as *n*_A_ and *n*_B_, and write their total as *n*. Let $\bar{\varepsilon}_{w}$ be the average strength of epistatic interactions for pairs of fixed mutations within each population, and $\bar{\varepsilon}_{b}$ the average strength for pairs of fixed mutations between (that is, in different) populations. We can then write:

$w_{H}\cong\exp\left\{ \alpha_{1}\bar{\varepsilon}_{b}n_{A}n_{B}-\left( \frac{1}{2}-\alpha_{1} \right)\frac{\bar{\varepsilon}_{w}}{2}\left( n_{A}^{2}-n_{A}+n_{B}^{2}-n_{B} \right) \right\}$

$=\exp\left\{ \bar{\varepsilon}_{b}\alpha_{1}F(1-F)n^{2}-\bar{\varepsilon}_{w}\left( \frac{1}{2}-\alpha_{1} \right)\left( \left( \frac{1}{2}-F(1-F) \right)n^{2}-\frac{n}{2} \right) \right\}$ $=\exp\left\{ \left( \bar{\varepsilon}_{b}+\bar{\varepsilon}_{w} \right)n^{2}F\left( 1-F \right)-\bar{\varepsilon}_{w}\binom{n}{2} \right\}$, (S11)

where *F* = *n*_A_ /*n* is the fraction of all substitutions that fixed in population A. This is the source of equation (2) in the main paper. The approximation in (S11) will be accurate when the variance in the epistatic effects is sufficiently small. (The exact value of $w_{H}$ requires integrating over the distributions of those effects, and for simplicity we choose not to do that here.)

Equation (S11) gives insights about how hybrid fitness evolves in the long run in two contrasting situations. The first insight applies to cases in which equal numbers of mutations have fixed in the two populations (*F* = ½). When the total number of mutations fixed is large, terms in the exponent of (S11) that involve *n*^2^ dominate. With symmetric divergence between the populations (*F* = ½), equation (S11) reduces to

$\bar{w}_{H}\cong\exp\left\{ \frac{1}{16}\left( \bar{\varepsilon}_{b}-\bar{\varepsilon}_{w} \right)n^{2}+\frac{1}{8}\bar{\varepsilon}_{w}n \right\}$. (S12)

The average epistatic fitness coefficients, $\bar{\varepsilon}_{b}$ and $\bar{\varepsilon}_{w}$, change as evolution proceeds in allopatry. We observe in our simulations that ultimately hybrid fitness always declines. That implies the quantity ($\bar{\varepsilon}_{b}$ – $\bar{\varepsilon}_{w}$) which appears in the exponent of equation (S12) is negative when the number of fixations is large. The explanation is that the average interaction between mutations fixed in different populations are similar to what it is for any two random mutations, which in our yeast dataset is $\bar{\varepsilon}$ = -5 x 10^-7^, whose negative value implies declining hybrid fitness as parental populations diverge. By contrast, interactions between mutations fixed within populations have been vetted by selection, and are therefore typically less deleterious, or even positive. The simulations show that after 50 mutations have fixed in a population, $\bar{\varepsilon}_{w}$ is 0.00667. In consequence, ($\bar{\varepsilon}_{b}$ – $\bar{\varepsilon}_{w}$) is negative, which explains why hybrids ultimately become less fit.

Equation (S11) also gives insight into hybrid viability when evolution in the two populations is highly asymmetric (*F* << 1). In that event, hybrid fitness is simply

$w_{H}\cong\exp\left\{ -\frac{\bar{\varepsilon}_{w}}{4}\binom{n}{2} \right\}$. (S12)

We estimate that $\bar{\varepsilon}_{w}$ = 1.04x10^-3^ (mean after 100 total fixed substitutions, σ=3.9x10^-4^) within our simulations based on the empirical yeast data, and so we expect that hybrid fitness will decrease rapidly in time when the two populations are evolving at very different rates.

A final comment about equation (S11) is that it clarifies the relationship between our model and that introduced by Orr [13-15]. He implicitly assumed that $\bar{\varepsilon}_{w}$ = -$\bar{\varepsilon}_{b}$ that is, the strength of positive epistasis between co-adapted alleles within a population is equal to the strength of the negative effects of introducing new interactions. Under these conditions, (S11) reduces to:

$w_{H}\cong\exp\left\{ \frac{1}{4}\bar{\varepsilon}\binom{n}{2} \right\}$*.* (S13)

This expression is equivalent to Orr’s original model [13].

2.b *The Trajectory of Hybrid Fitness through time*

To gain further insight to how hybrid fitness changes in time, we return to the case where equal numbers of mutations have been fixed in the two populations. For simplicity, we begin by assuming that the mean effect of epistatic interactions between mutations fixed in different populations, $\bar{\varepsilon}_{b}$, are equal to those between mutations fixed within a population, $\bar{\varepsilon}_{w}$. Then (S11) can be written as:

$w_{H}\cong\exp\left\{ \frac{1}{4}\left( \frac{\bar{\varepsilon}_{n}n^{2}}{2}-\bar{\varepsilon}_{n}\binom{n}{2} \right) \right\}=\exp\left\{ \frac{\bar{\varepsilon}_{n}}{8}n \right\}$, (S14)

where $\bar{\varepsilon}_{n}$ is the mean epistatic effect after *n* mutations have been fixed. If $\bar{\varepsilon}_{n}$ does not depend on *n*, then there is a constant proportional decrease in hybrid fitness with each additional mutation fixed. Hybrid fitness does not decline at an accelerating rate: no “snowball” effect occurs.

A constant rate of decline in hybrid fitness is no longer expected if $\bar{\varepsilon}_{n}$ varies with *n*. To understand the consequences, assume a simple linear model for how $\bar{\varepsilon}_{n}$ varies with *n*:

$\bar{\varepsilon}_{n}=\alpha+\beta n$*.*  (S15)

This linear model is empirically plausible, given the experimental yeast data indicating such a relationship where $\beta$is estimated to be negative. Assuming that mutations are fixed in decreasing order of their direct fitness effects, we estimate *α* = -5x10^-3^ and β = -6x10^-7^. Then (S14) can be written as:

$w_{H}\cong\exp\left\{ \frac{1}{2}(\alpha n+\beta n^{2}) \right\}$. (S16)

Under these assumptions, the rate of hybrid fitness decay “snowballs” if β is negative, meaning that epistatic interactions become increasingly deleterious. This is a fundamentally different mechanism for the snowball effect seen in previous models [13, 14, 17], which is based on increasing numbers of interactions.

2.c *Heterosis*

The most important difference between our model and previous work is the possibility of heterosis. There are three ways heterosis can occur in our model.

As discussed earlier, the interactions between mutations fixed in different populations have not been filtered by selection and so are expected on average to be deleterious. This implies that hybrid fitness will decline in the long term. Again assuming the simplified model of equation (S20), this implies *β* is negative. In the short term, however, heterosis occurs if α is positive and the total number of mutations fixed is less than –*α* / *β*. The yeast data suggest, however, that both *α*and *β* are negative. In this case, hybrids will on average show reduced fitness at all times.

Second, heterosis can occur if the strengths of epistasis between mutations fixed within a population differs from that between mutations fixed in different populations. Heterosis is expected to occur when (S11) is greater than 1, which requires

$n<\frac{2\bar{\varepsilon}_{w}}{\bar{\varepsilon}_{b}-\bar{\varepsilon}_{w}}$ *.* (S17)

Since $\bar{\varepsilon}_{b}$ is on average expected to be negative, and $\bar{\varepsilon}_{w}$ positive, we again do not, in general, expect heterosis. The value on the right hand of (S14) represents potential for heterosis. We find in simulations based on the yeast data that this value consistently converges to -1.6 ± 0.4 following 100 fixed substitutions, indicating that on average we expect hybrid fitness to decline in the long run.

A third way in which heterosis can occur is seen in our simulations. Occasionally, mutations that become fixed in different populations fortuitously have positive epistatic interactions. This causes hybrids to have high fitness. In the long term, however, as more mutations become fixed the average epistatic fitness effect prevails and hybrid fitness declines. In short, although on average we expect declining hybrid fitness as populations diverge, there is variance around that average that results in transient heterosis in a subset of populations.

2.d *Haldane's Rule*

Haldane's Rule is the observation that hybrid fitness is often more severely reduced in the heterogametic sex (males with XY sex determination, females with ZW sex determination) [1, 5, 6]. The leading explanation for Haldane's Rule is that because the X is hemizygous in males, alleles on the X that have recessive deleterious interactions with alleles on autosomes reduce hybrid male fitness [9]. To simplify calculations, we assume that direct fitness effects are completely additive (*h_i_* = ½). Nevertheless, Haldane’s Rule does emerge from our model under some conditions that we will now explore. Here we assume XY sex determination, but the results apply to ZW systems if we substitute Z for X, W for Y, and interchange male and female.

We make a distinction between substitutions at autosomal loci and the sex chromosomes. Let 𝕏_A_, 𝕐_A_, and 𝔸_A_ be respectively the set of fixed in population A on the X, the Y, and the autosomes. The corresponding sets of mutations fixed in population B are denoted with the subscript B. Consider hybrids whose mothers are from population A and fathers from population B. To simplify the math, assume that *h_i_*=1/2, *α*_1_=1/4, *α*_2_=1/2. We define H_AB_ as the ratio of the mean hybrid male fitness to mean hybrid female fitness. Again assuming the fitness function given by (1), that all selection coefficients are much smaller than 1 and that dominance is completely additive, that ratio is:

$H_{\mathrm{AB}}\cong\exp\left\{ \frac{1}{2}\left( \sum_{i\in\mathbb{Y}_{A}} s_{i}-\sum_{i\in\mathbb{X}_{A}} s_{i} \right)+\frac{1}{8}\left( \sum_{i,j\in\mathbb{Y}_{A}} \varepsilon_{ij}-\sum_{i,j\in\mathbb{X}_{A}} \varepsilon_{ij} \right)+\frac{1}{4}\left( \sum_{i\in\mathbb{Y}_{A},j\in\mathbb{A}_{A}} \varepsilon_{ij}-\sum_{i\in\mathbb{X}_{A},j\in\mathbb{A}_{A}} \varepsilon_{ij} \right)+\frac{1}{4}\left( \sum_{i\in\mathbb{Y}_{A},j\in\mathbb{A}_{B}} \varepsilon_{ij}-\sum_{i\in\mathbb{X}_{A},j\in\mathbb{A}_{B}} \varepsilon_{ij} \right)+\frac{1}{4}\left( \sum_{i\in\mathbb{Y}_{A},j\in\mathbb{X}_{B}} \varepsilon_{ij}-\sum_{i\in\mathbb{X}_{A},j\in\mathbb{X}_{B}} \varepsilon_{ij} \right) \right\}.$ (S18)

The sets of terms in the first three parentheses are direct and epistatic fitness effects of the Y and X with mutations in population A. If males and females have equal mean fitness, these terms cancel out and H_AB_ becomes:

$H_{AB}\cong\exp\left\{ \frac{1}{4}\left( \sum_{i\in\mathbb{Y}_{A},j\in\mathbb{A}_{B}} \varepsilon_{ij}-\sum_{i\in\mathbb{X}_{A},j\in\mathbb{A}_{B}} \varepsilon_{ij} \right)+\left( \sum_{i\in\mathbb{Y}_{A},j\in\mathbb{X}_{B}} \varepsilon_{ij}-\sum_{i\in\mathbb{X}_{A},j\in\mathbb{X}_{B}} \varepsilon_{ij} \right) \right\}$. (S19)

Haldane’s Rule results when this quantity is less than 1, that is, when the exponent on the right side is negative. The exponent is a sum of four terms. The first two terms represent the epistatic interactions between the sex chromosomes of population A and the autosomes of population B. If interactions tend to be deleterious, Haldane’s rule holds when the interactions with the X are less deleterious than those with the Y. If interactions are thought to be positive, Haldane’s rule would be a result of more interactions between the X and the autosomes. The second two terms represent the interactions between the X of population B and the sex chromosomes of population A. If there are more deleterious interactions between the X and Y than between two X’s, then Haldane’s Rule is once again expected. This may be the case if mutations fixed on the sex chromosomes are sexually antagonistic epistatically. That is, mutations fixed on the X may synergize to increase female fitness, but interact deleteriously with mutations fixed on the Y.

In sum, our model shows that Haldane’s Rule can result from certain patterns of epistatic fitness effects even in the absence of strong dominance. This expands the range of conditions leading to the rule beyond the standard hypothesis.

2.e *Cyto-Nuclear Interactions*

Interactions between the cytoplasm (including the mitochondria) and nuclear genes can have important effects on hybrid fitness [5]. Here we ask if these interactions are expected to be an important component of speciation under our model.

Denote the fitness of hybrids with a father from population A and a mother from population B as $\bar{W}_{AB}$; the fitness of hybrids from the reciprocal cross is $\bar{W}_{BA}$. Let 𝔸_A_ and
$\mathbb{C}_{A}$ be respectively the sets of mutations that have fixed on the autosomes and in the cytoplasm in population A; in the analogous quantities for population B, the subscripts are replaced by B. We again assume that *h_i_*=1/2, *α*_1_=1/4, *α*_2_=1/2. Averaging over the two reciprocal crosses, we find from fitness function (1) that the relative fitness of hybrids is

$\bar{w}_{H}=\frac{\bar{W}_{AB}+\bar{W}_{BA}}{\bar{W}_{AA}+\bar{W}_{BB}}=\exp\left\{ \left( \sum_{i\in\mathbb{C}_{A},j\in\mathbb{A}_{B}} \varepsilon_{ij}-\sum_{i\in\mathbb{C}_{A},j\in\mathbb{A}_{A}} \varepsilon_{ij} \right)+\left( \sum_{i\in\mathbb{C}_{B},j\in\mathbb{A}_{A}} \varepsilon_{ij}-\sum_{i\in\mathbb{C}_{B},j\in\mathbb{A}_{B}} \varepsilon_{ij} \right)+\left( 2\sum_{i\in\mathbb{A}_{B},j\in\mathbb{A}_{A}} \varepsilon_{ij}-\sum_{i,j\in\mathbb{A}_{A}} \varepsilon_{ij}-\sum_{i,j\in\mathbb{A}_{B}} \varepsilon_{ij} \right) \right\}.$ (S20)

The exponent is a sum of seven terms. Hybrids will have lower fitness than parental individuals when their total is negative. The final three terms are dependent entirely on nuclear mutations. Cytoplasmic mutations can contribute to speciation in several ways distinct from nuclear ones. The first two sets of parentheses include interactions between nuclear and cytoplasmic mutations. Within each parentheses, the first term includes interactions between the cytoplasmic mutations and nuclear mutations between populations, whereas the second represents the cyto-nuclear interactions within each population. Unlike nuclear-nuclear interactions, however, hybrid fitness is not affected by breakdown of interactions within the cytoplasmic genes. Our model therefore suggests that cyto-nuclear incompatibilities are only likely to play a significant role in speciation when many cytoplasmic mutations become fixed, increasing the importance of the first four terms of (S20).

2.f *Darwin’s Corollary*

Many empirical studies find that crosses produce asymmetric hybrid fitnesses depending on which species is paternal and which is maternal. This pattern, termed Darwin’s Corollary to Haldane’s Rule, is thought to result from uniparentally inherited genetic elements (sex chromosomes and the cytoplasm, including mitochondria) [5, 6]. In this section we show how Darwin’s Corollary emerges in the framework of our model. We begin with the case of sex chromosomes, then consider cytoplasmic effects.

Consider a species with XY sex determination. (As in the earlier section on Haldane’s Rule, the results are easily translated so as to apply to ZW systems.) Again appealing to the fitness function (1), we find that the fitness of a hybrid male with a father from population A and mother from population B, labeled $\bar{W}_{AB}^{m}$, relative to a hybrid male of the reciprocal cross is

$\frac{\bar{W}_{AB}^{m}}{\bar{W}_{BA}^{m}}\cong\exp\left\{ \begin{aligned} \frac{1}{4}\left( 2\sum_{i\in\mathbb{Y}_{A}} s_{i}+\frac{1}{2}\sum_{i,j\in\mathbb{Y}_{A}} \varepsilon_{ij}+\sum_{i\in\mathbb{Y}_{A},j\in\mathbb{A}_{A}} \varepsilon_{ij} \right)-\frac{1}{4}\left( 2\sum_{i\in\mathbb{X}_{A}} s_{i}+\frac{1}{2}\sum_{i,j\in\mathbb{X}_{A}} \varepsilon_{ij}+\sum_{i\in\mathbb{X}_{A},j\in\mathbb{A}_{A}} \varepsilon_{ij} \right)+ \\ \frac{1}{4}\left( 2\sum_{i\in\mathbb{X}_{B}} s_{i}+\frac{1}{2}\sum_{i,j\in\mathbb{X}_{B}} \varepsilon_{ij}+\sum_{i\in\mathbb{X}_{B},j\in\mathbb{A}_{B}} \varepsilon_{ij} \right)-\frac{1}{4}\left( 2\sum_{i\in\mathbb{Y}_{B}} s_{i}+\frac{1}{2}\sum_{i,j\in\mathbb{Y}_{B}} \varepsilon_{ij}+\sum_{i\in\mathbb{Y}_{B},j\in\mathbb{A}_{B}} \varepsilon_{ij} \right)+ \\ \frac{1}{4}\left( \sum_{i\in\mathbb{Y}_{A},j\in\mathbb{A}_{B}} \varepsilon_{ij}+\sum_{i\in\mathbb{X}_{B},j\in\mathbb{A}_{A}} \varepsilon_{ij}+\sum_{i\in\mathbb{Y}_{A},j\in\mathbb{X}_{B}} \varepsilon_{ij} \right)-\frac{1}{4}\left( \sum_{i\in\mathbb{Y}_{B},j\in\mathbb{A}_{A}} \varepsilon_{ij}+\sum_{i\in\mathbb{X}_{A},j\in\mathbb{A}_{B}} \varepsilon_{ij}+\sum_{i\in\mathbb{Y}_{B},j\in\mathbb{X}_{A}} \varepsilon_{ij} \right) \end{aligned} \right\}$. (S21)

Darwin’s Corollary follows whenever this quantity differs from 1, a condition that holds when the sum of the terms in the exponent is not exactly zero. In general, there is no biological (or mathematical) reason to expect that condition to occur, and so Darwin’s Corollary is expected as a generic result of divergent evolution of sex chromosomes in allopatry.

The biological meaning of terms in equation (S21) are as follows. The first two sets of terms in parentheses capture direct and epistatic fitness effects of the mutations fixed on the Y and X in populations A, respectively. The third and fourth sets of terms within parentheses capture the direct and epistatic fitness effects of mutations fixed on the X and Y in population B. If mean male and female fitnesses are equal in both populations, these terms cancel out, leaving the following expression:

$\frac{\bar{W}_{AB}^{m}}{\bar{W}_{BA}^{m}}\cong\exp\left\{ \begin{aligned} 1/4\left( \sum_{i\in\mathbb{Y}_{A},j\in\mathbb{A}_{B}} \varepsilon_{ij}+\sum_{i\in\mathbb{X}_{B},j\in\mathbb{A}_{A}} \varepsilon_{ij}+\sum_{i\in\mathbb{Y}_{A},j\in\mathbb{X}_{B}} \varepsilon_{ij} \right)- \\ 1/4\left( \sum_{i\in\mathbb{Y}_{B},j\in\mathbb{A}_{A}} \varepsilon_{ij}+\sum_{i\in\mathbb{X}_{A},j\in\mathbb{A}_{B}} \varepsilon_{ij}+\sum_{i\in\mathbb{Y}_{B},j\in\mathbb{X}_{A}} \varepsilon_{ij} \right) \end{aligned} \right\}.$ (S22)

There are three sets of relationships remaining: the epistatic effects of between the Y of one population and the autosomes of the other, the epistatic effects of the X of one population and the autosomes of the other, and epistatic interactions between the sex chromosomes of different populations. Each of these components is likely to have a non-zero value whenever the populations evolve at different rates. As an example, consider the case when only one population has fixed mutations on the Y. The terms of the first bracket will then immediately be non-zero, contributing to Darwin’s Corollary. Hybrid females do not experience any of these effects. Under our assumption that the parental populations are fixed for their mutations, *F*_1_ females produced by the reciprocal crosses have identical genotypes and so there is no asymmetry in their fitness depending on the cross.

Finally, we consider the contribution that cytoplasmic factors, such as mitochondria or chloroplasts, can make towards the Corollary. Consider the fitness of hybrids with a mother from population A and a father from B relative to the fitness of hybrids from the reciprocal cross. Continuing with the notation from the previous section, that ratio is**:**

$\frac{\bar{W}_{AB}}{\bar{W}_{BA}}\cong\exp\left\{ \frac{1}{4}\left( \begin{aligned} \left( 2\sum_{i\in\mathbb{C}_{B}} s_{i}+\frac{1}{2}\sum_{i,j\in\mathbb{C}_{B}} \varepsilon_{ij}+\sum_{i\in\mathbb{C}_{B},j\in\mathbb{A}_{A}} \varepsilon_{ij}+\sum_{i\in\mathbb{C}_{B},j\in\mathbb{A}_{A}} \varepsilon_{ij} \right)- \\ \left( 2\sum_{i\in\mathbb{C}_{A}} s_{i}+\frac{1}{2}\sum_{i,j\in\mathbb{C}_{A}} \varepsilon_{ij}+\sum_{i\in\mathbb{C}_{A},j\in\mathbb{A}_{A}} \varepsilon_{ij}+\sum_{i\in\mathbb{C}_{A},j\in\mathbb{A}_{B}} \varepsilon_{ij} \right) \end{aligned} \right) \right\}.$ (S23)

Darwin’s Corollary is realized whenever this ratio is not exactly 1, which occurs when the total of the sum of terms in the exponent is not exactly zero. Again, under generic circumstances we expect the Corollary to be realized. The set of terms in the first pair of parentheses represent the direct and epistatic fitness interactions between mutations fixed in cytoplasm B with all other mutations carried by hybrids. The second set of parentheses contains the corresponding fitness effects of mutations in cytoplasm A. If different mutations have fixed in the two cytoplasms, we expect these two sets of terms to be unequal, with the result that hybrids from the reciprocal crosses will have different fitnesses.

If the parental populations have equal fitnesses, all but the last two summations in (S23) cancel out. We are left with a simplified expression:

$\frac{\bar{W}_{AB}}{\bar{W}_{BA}}\cong\exp\left\{ \frac{1}{4}\sum_{i\in\mathbb{C}_{B},j\in\mathbb{A}_{A}} \varepsilon_{ij}-\frac{1}{4}\sum_{i\in\mathbb{C}_{A},j\in\mathbb{A}_{B}} \varepsilon_{ij} \right\}.$ (S24)

Each of the two summations in (S23) represents the total fitness effect of epistatic interactions between the cytoplasm of one population and the nuclear genes of the other. If these interactions are not symmetric between the populations, either because the effects of individual mutations differ on average or because there have been different numbers of mutations fixed in the two populations, then Darwin’s Corollary can again result. In general there is no biological reason to expect perfect symmetry in epistatic effects or substitution rates, therefore some hybrid asymmetry should be the default expectation.

**S1 References**

1. Haldane JB. Sex ratio and unisexual sterility in hybrid animals. Genetics. 1922;12:101-9.

2. Schilthuizen M, Giesbers M, Beukeboom L. Haldane's rule in the 21st century. Heredity (Edinb). 2011;107:95-102.

3. Turelli M, Orr HA. The dominance theory of Haldane's rule. Genetics. 1995;140:389-402.

4. Bolnick DI, Turelli M, Lopez-Fernandez H, Wainwright PC, Near TJ. Accelerated mitochondrial evolution and "Darwin's corollary": asymmetric viability of reciprocal F1 hybrids in centrarchid fishes. Genetics. 2008;178:1037-48.

5. Brandvain Y, Pauly GB, May MR, Turelli M. Explaining Darwin’s corollary to Haldane’s rule: the role of mitonuclear interactions in asymmetric postzygotic isolation among toads. Genetics. 2014;197:743-7.

6. Turelli M, Moyle LC. Asymmetric postmating isolation: Darwin's corollary to Haldane's rule. Genetics. 2007;176:1059-88.

7. Costanzo M, VanderSluis B, Koch EN, Baryshnikova A, Pons C, Tan G, et al. A global genetic interaction network maps a wiring diagram of cellular function. Science. 2016;353.

8. Coyne JA, Orr HA. Speciation. Sunderland, MA: Sinauer Associates; 2004.

9. Muller HJ. Bearing of the *Drosophila* work on systematics. In: Huxley J, editor. The New Systematics. Oxford, UK: Oxford University Press; 1940. p. 185-268.

10. Lee HY, Chou JY, Cheong L, Chang NH, Yang SY, Leu JY. Incompatibility of nuclear and mitochondrial genomes causes hybrid sterility between two yeast species. Cell. 2008;135:1065-73.

11. Trier CN, Hermansen JS, Saetre G-P, Bailey RI. Evidence for Mito-Nuclear and Sex-Linked Reproductive Barriers between the Hybrid Italian Sparrow and Its Parent Species. PLoS Genet. 2014;10:e1004075.

12. Livingstone K, Olofsson P, Cochran G, Dagilis A, MacPherson K, Seitz KA. A stochastic model for the development of Bateson–Dobzhansky–Muller incompatibilities that incorporates protein interaction networks. Math Biosci. 2012;238:49-53.

13. Orr HA. The population genetics of speciation: the evolution of hybrid incompatibilities. Genetics. 1995;139:1805-13.

14. Orr HA, Turelli M. The evolution of postzygotic isolation: accumulating Dobzhansky-Muller incompatibilities. Evolution. 2001;55:1085-94.

15. Turelli M, Orr HA. Dominance, epistasis, and the genetics of postzygotic isolation. Genetics. 2000;154:1663-79.

16. Welch JJ. Accumulating Dobzhansky-Muller incompatibilities: reconciling theory and data. Evolution. 2004;58:1145-56.

17. Orr HA, Presgraves DC. Speciation by postzygotic isolation: Forces, genes and molecules. Bioessays. 2000;22:1085-94.
