# Supplementary figures and images for: The evolution of hybrid fitness during speciation
Source: PLoS Genet. 2019 May 6;15(5):e1008125. doi: 10.1371/journal.pgen.1008125 (PMC6502311; doi:10.1371/journal.pgen.1008125)

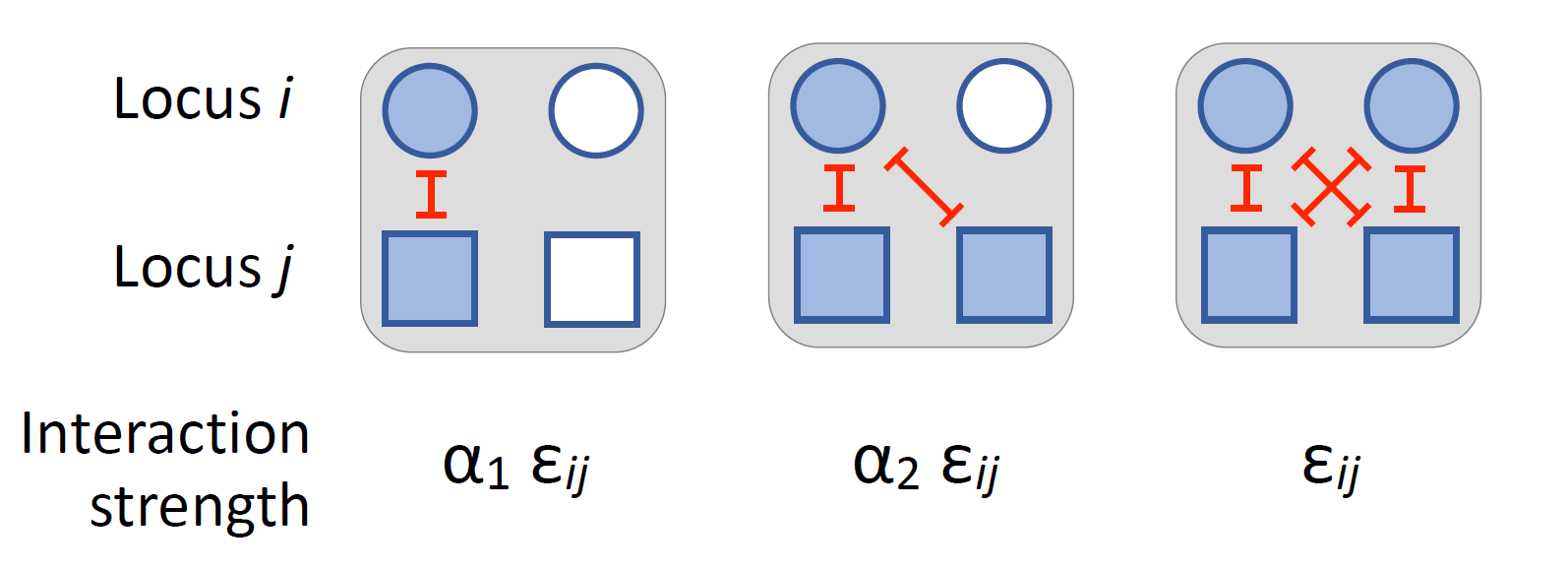

Supplement: S1 Fig — Schematic of the 3 possible strengths of epistatic interactions when (from left to right) both loci are heterozygous for derived (shaded) allele, one is heterozygous and one is homozygous, and both are homozygous. The red lines show the epistatic interactions. These values correspond to Turelli and Orr’s notation of H0, H1, H2 from left to right, respectively. (TIF) [file pgen.1008125.s002.tif]

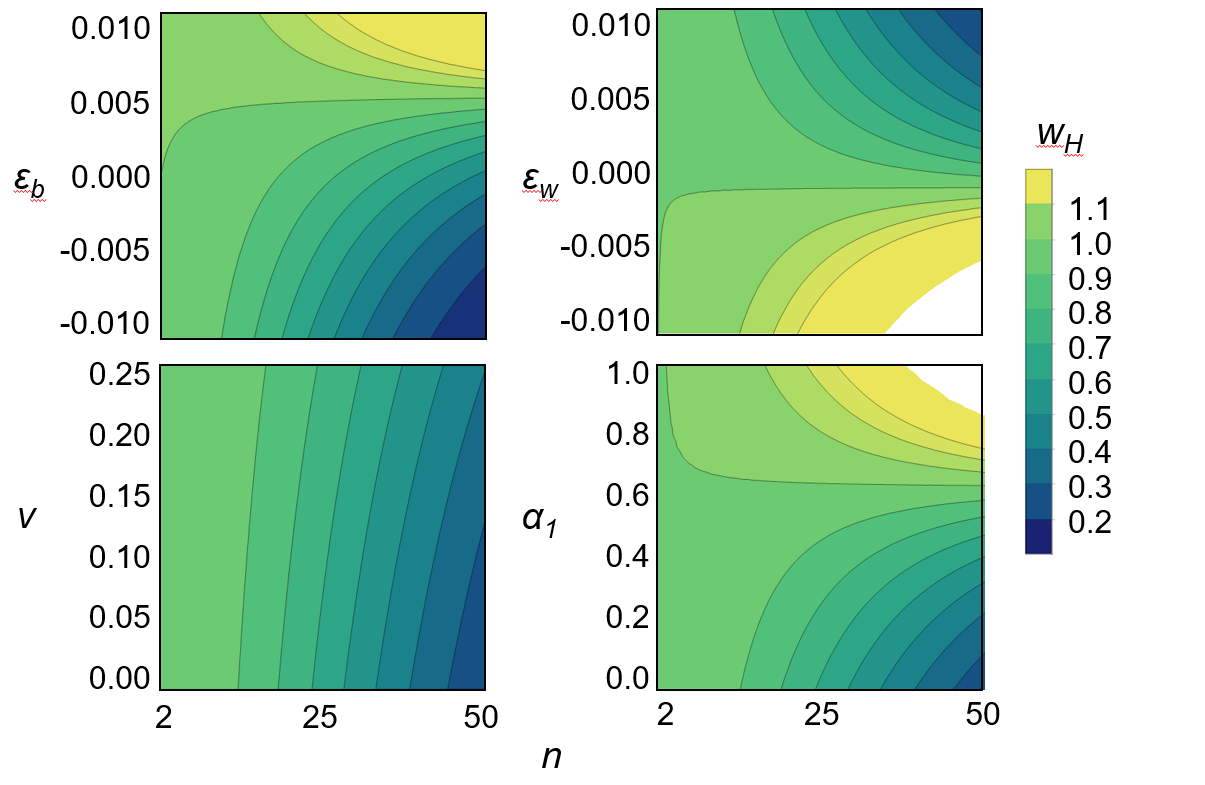

Supplement: S2 Fig — The effect of varying the strength of epistasis, asymmetry in divergence and dominance of epistasis to hybrid fitness. All heat maps are numerical evaluations of hybrid fitness given by Eq (2) of the main text, with default parameter values ε¯b = -0.005, ε¯w = 0.01, v = 1/4, α1 = 1/4. Varying ε¯b (top left) causes hybrid fitness to decline more rapidly when between population epistasis becomes more deleterious, and heterotic when they are positive. The inverse pattern is observed for ε¯w (top right). Increasing asymmetry (lower v values, bottom left) decreases hybrid fitness more rapidly, while increasing epistatic dominance (bottom right) retains within population epistasis causing heterosis (since |ε¯b|<|ε¯b|), while lower dominance causes more rapid speciation. (TIF) [file pgen.1008125.s003.tif]

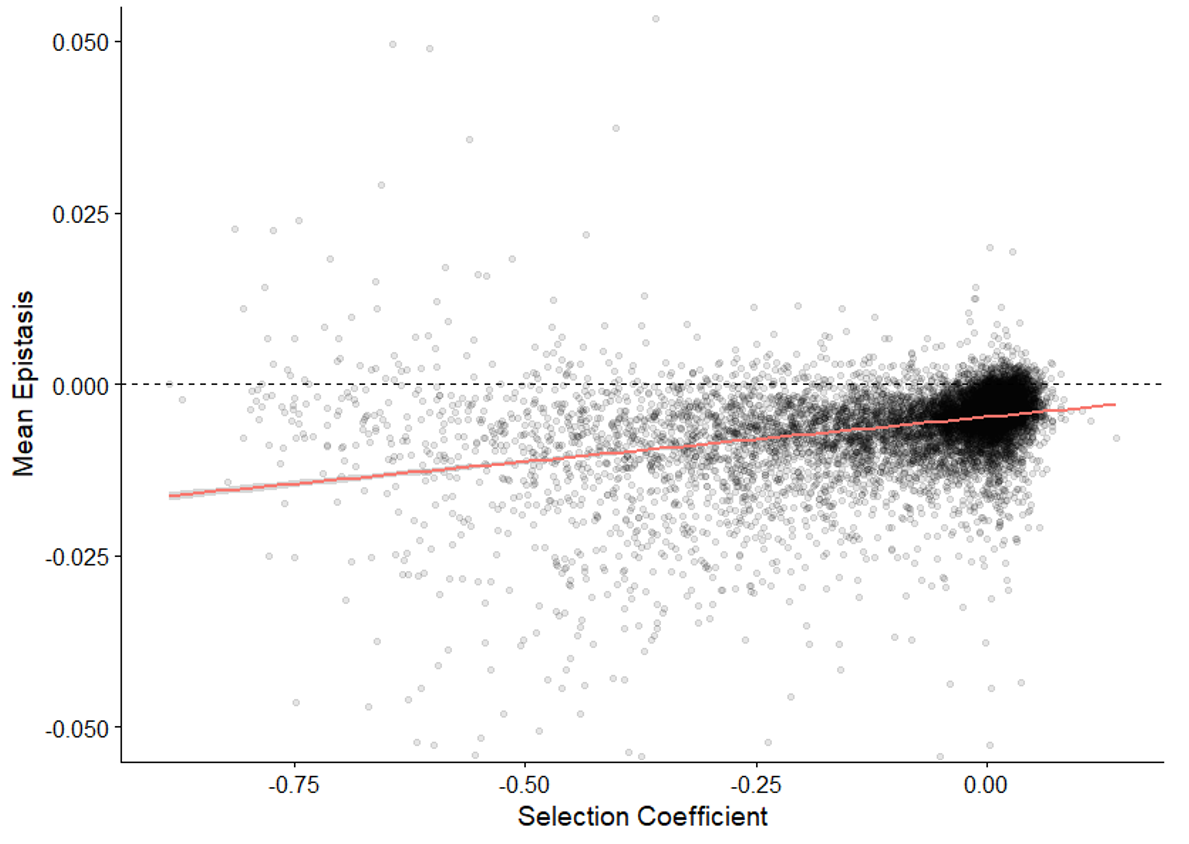

Supplement: S3 Fig — In yeast knockouts, the mean epistatic effect of mutations correlates with the mutations’ direct fitness effects. Each point is one of the mutations in the yeast epistatic interaction data-set. The X-axis is the direct selection coefficient observed in lab experiments, while the Y-axis is the average of all epistatic interactions involving that mutation with all other genes. The red line is the linear fit to the data (adjusted R-squared of 0.103, p<10−16). (TIF) [file pgen.1008125.s004.tif]

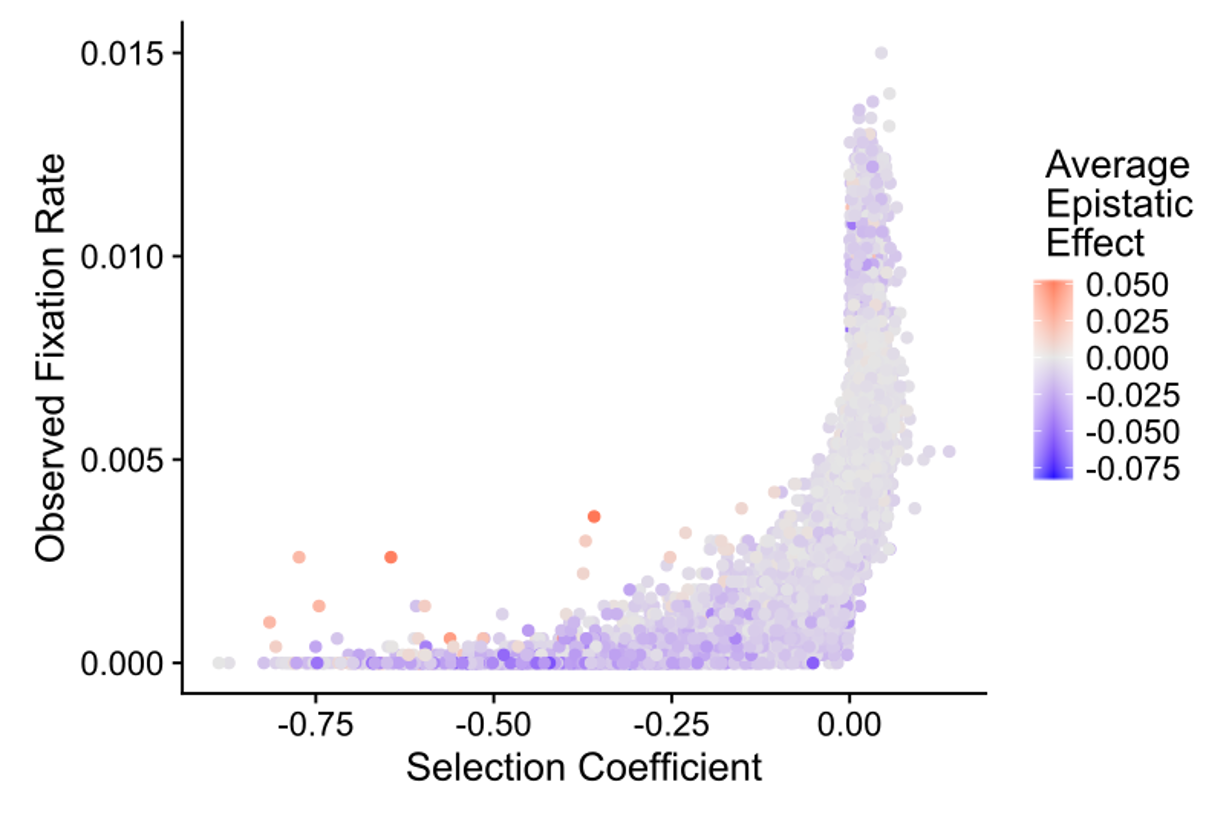

Supplement: S4 Fig — Observed simulation fixation rates for the 10588 mutations in the yeast epistatic network dataset. Points in red are mutations with on average positive epistasis, while points in blue are epistatically neutral or deleterious. Mutations with even highly negative direct selection coefficients are fixed frequently when their epistatic effects are largely positive. (TIF) [file pgen.1008125.s005.tif]

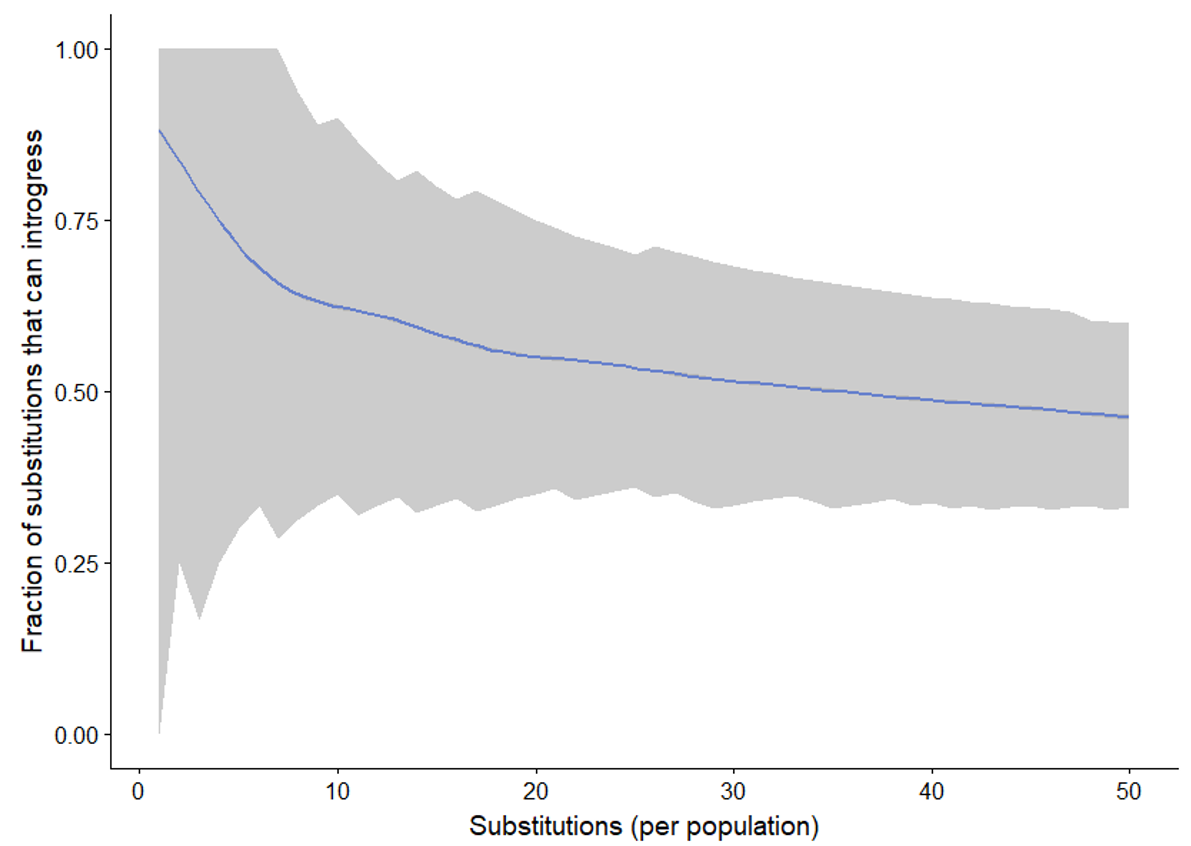

Supplement: S5 Fig — The fraction of substitutions in one population that are positively selected in the other population over the course of the simulation. Blue line is the mean of 10000 simulations, grey area is the 95% confidence interval. Nearly all substitutions fixed early are beneficial in the ancestral background and so will fix in both populations if gene flow allows the derived allele to cross between populations. As divergence continues, fewer and fewer of the substitutions in one population are positively selected in the genomic background of the other population. The proportion of positively selected mutations remains high even when hybrid fitness is expected to be very low (<0.2, Fig 4). (TIF) [file pgen.1008125.s006.tif]

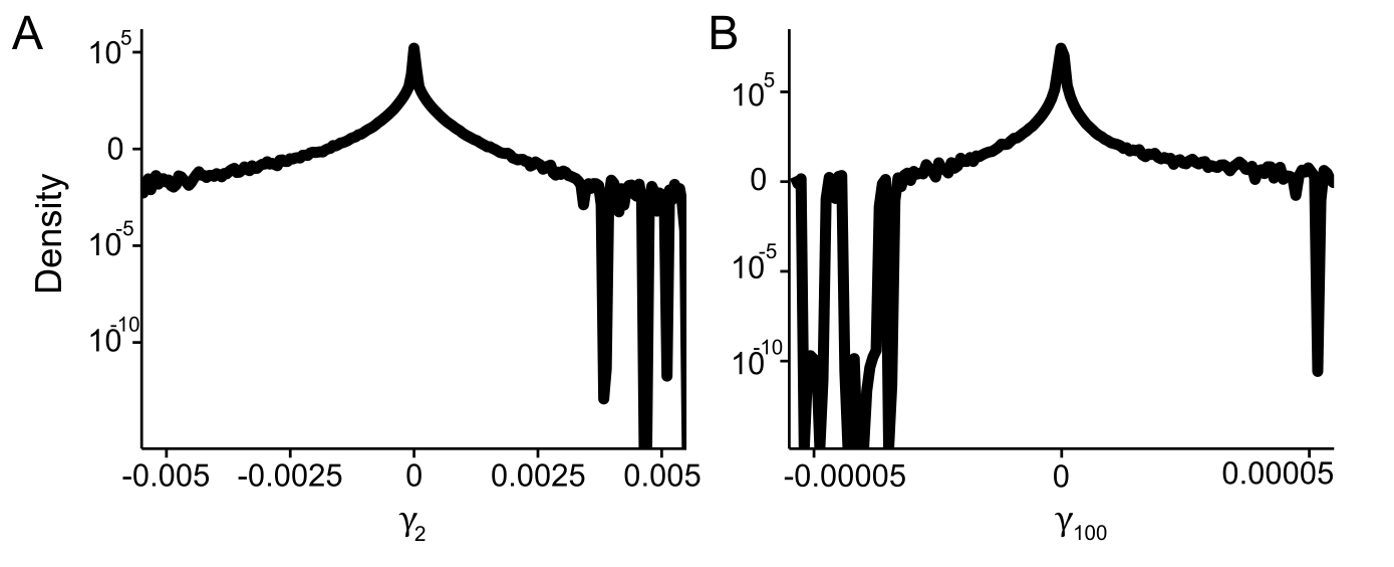

Supplement: S6 Fig — The distribution of γ2 (A) and γ100 (B) values, representing the potential of each individual interaction to contribute to speciation after a total of 2 and 100 mutations have been fixed in two independently evolving populations. Since γ is a cross product of the frequency an interaction is seen in a hybrid and its fitness effects in that hybrid, positive values represent potential for heterosis, while negative ones represent reproductive isolation. The vast majority of interactions are unlikely to contribute to either process. (TIF) [file pgen.1008125.s007.tif]

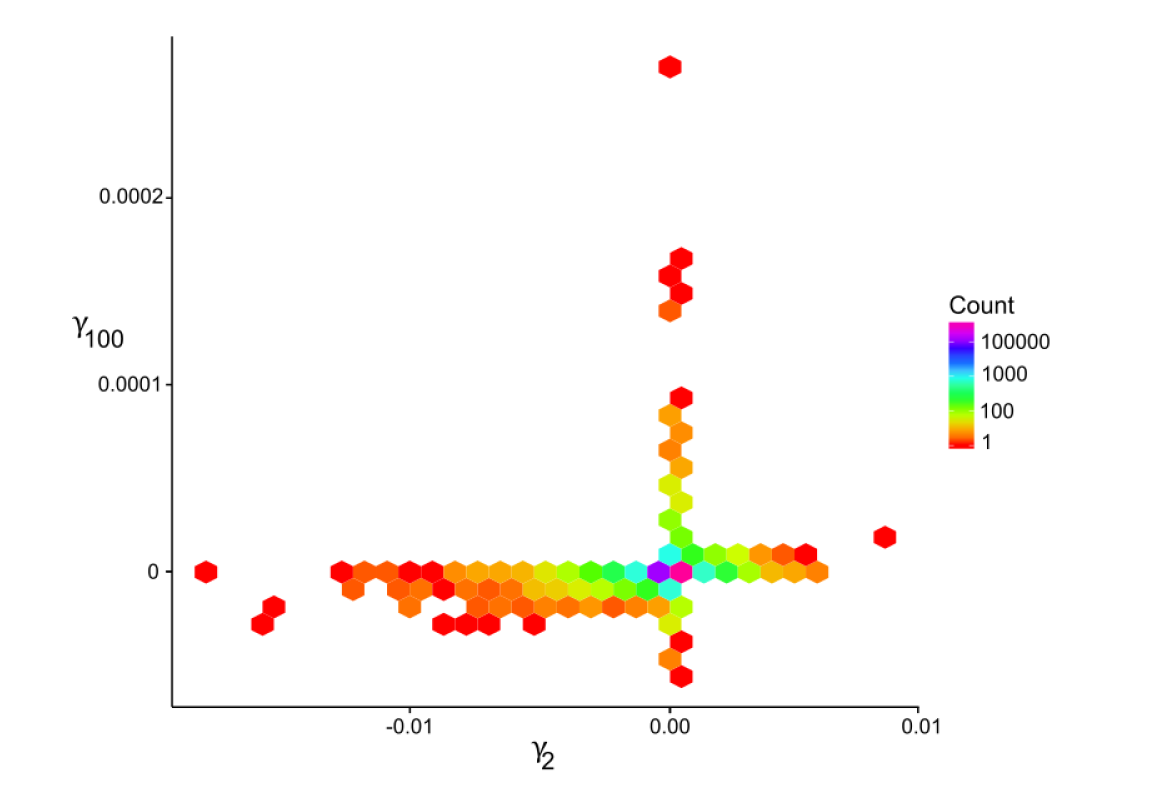

Supplement: S7 Fig — The relationship between γ2 and γ100 for all interactions. Most interactions have no effect towards hybrid speciation either early or late in the process of speciation. The interactions that are most important early in the process (large absolute γ2) do not remain important as the populations continue to diverge. This suggest that different interactions may be contributing to hybrid fitness at different times during the process of speciation. (TIF) [file pgen.1008125.s008.tif]

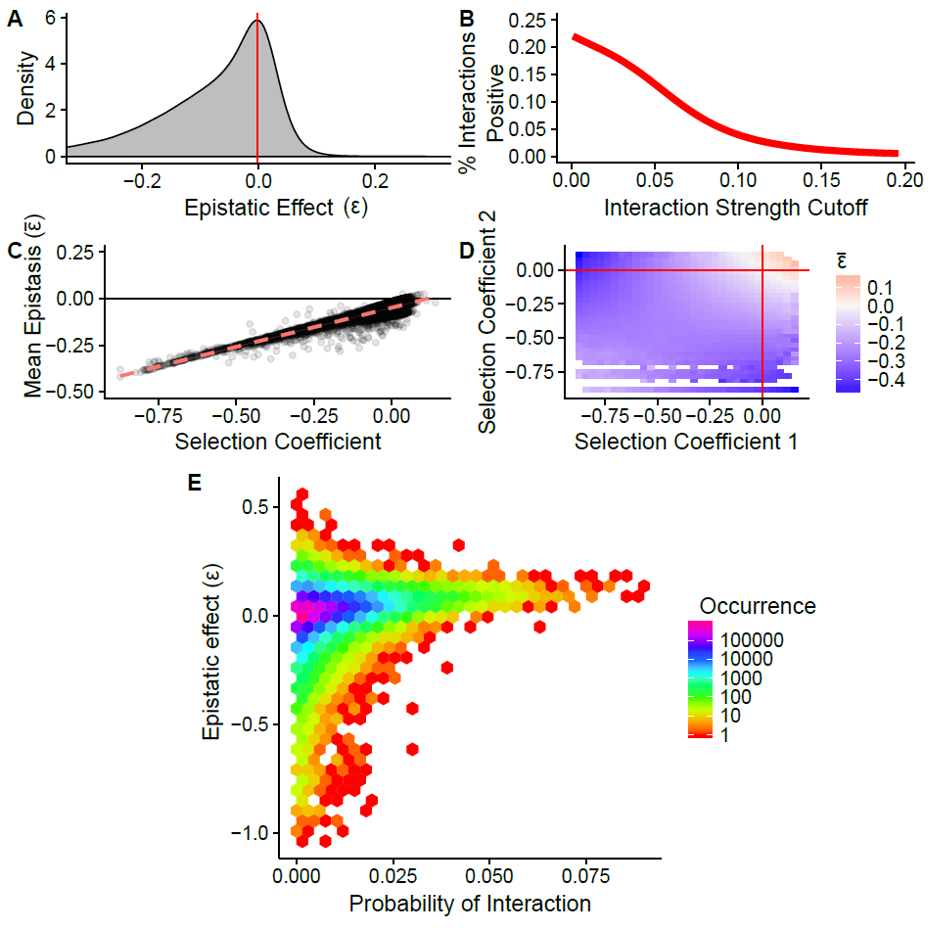

Supplement: S8 Fig — Summary of network using additive epistasis measures. A) The distribution of epistatic effects. B) The proportion of positive epistatic interactions as a function of the cutoff of the absolute value of the interaction. Additive measures of epistasis lead to far fewer positive interactions of large effect, reducing the proportion of positive interactions in the network to negligible amounts if strong cutoffs are considered. C) The highly linear relationship between additive epistatic effect and direct fitness effects of mutations. D) The relationship in C is derived from the strong pattern of deleterious mutations having strongly deleterious interactions, while positively selected interactions tend to occur between positively selected mutations. E) Since positively selected mutations largely have positive interactions, the interactions likely to occur early in hybrids are on average positive. (TIF) [file pgen.1008125.s009.tif]

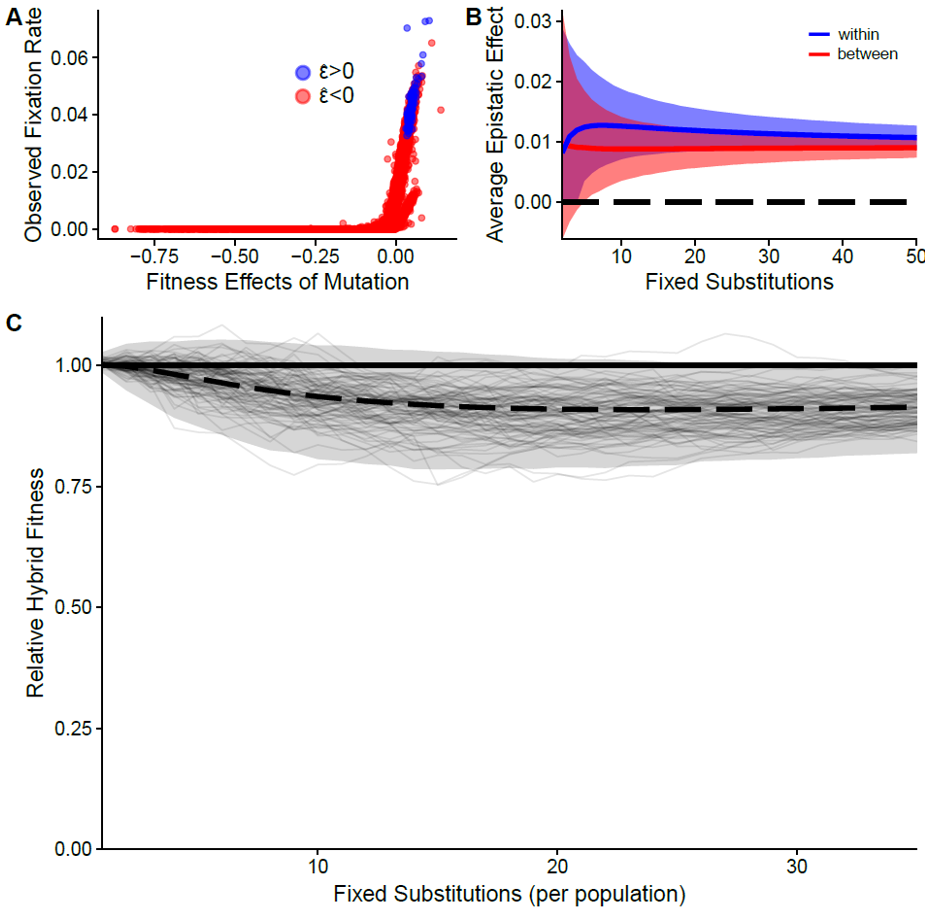

Supplement: S9 Fig — The simulation results for the additive epistatic model. (A) Under the additive model, only interactions of positive direct fitness effect tend to be fixed. (B) Since positively selected mutations share largely positive epistatic interactions, within and between population interactions slowly converge to the same values. (C) Hybrid fitness asymptotes under the additive model, since novel interactions first seen in hybrids are of roughly the same positive strength as the co-adapted interactions seen within populations (as seen in B). (TIF) [file pgen.1008125.s010.tif]

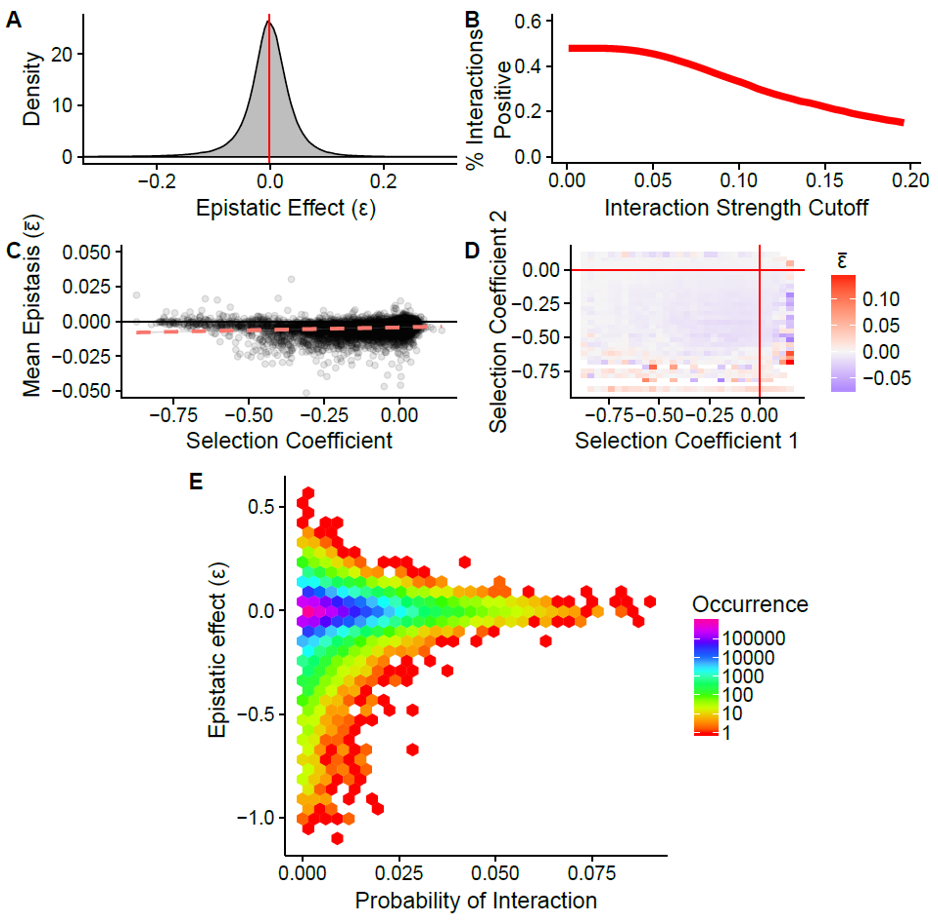

Supplement: S10 Fig — Results using the measures of epistasis presented in Costanzo et al. (2016). A) The distribution of epistatic effects. B) The proportion of positive epistatic interactions as a function of the cutoff of the absolute value of the interaction. C) The relationship between additive epistatic effect and direct fitness effects of mutations. The two are weakly but significantly correlated (r2 = ,p<10). D)The largest epistatic effects are seen between mutations of most extreme effects (both positive and negative). E) Interactions likely to occur early in speciation are of weaker and more positive effect than interactions that are unlikely to occur early on. (TIF) [file pgen.1008125.s011.tif]

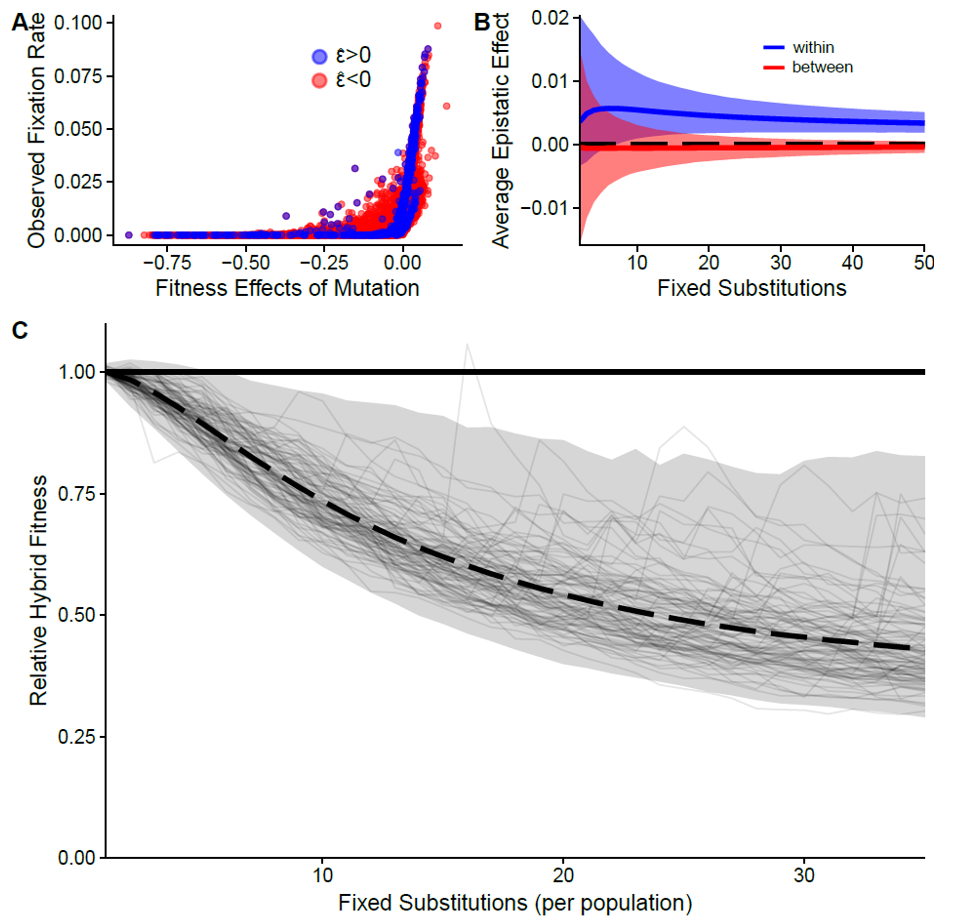

Supplement: S11 Fig — The simulation results for the Costanzo epistatic model. Results are qualitatively similar to the results presented in the main paper, with several exceptions. (A) The modified epistatic model results in mutations with extremely strong direct fitness effects rarely being fixed. Mutations with positive or negative epistatic effect are colored blue and red, respectively. (B) Epistatic effects between populations (red) converge to the average epistatic effect between populations as substitutions are fixed, while interactions within each population (blue) quickly become positive in the majority of the simulations. The curves capture the range of 95% of the simulations. (C) Hybrid fitness decreases more slowly than in the multiplicative case, but much faster than in the additive model. Dashed line represents simulation mean; faint grey lines give representative simulation contours. (TIF) [file pgen.1008125.s012.tif]

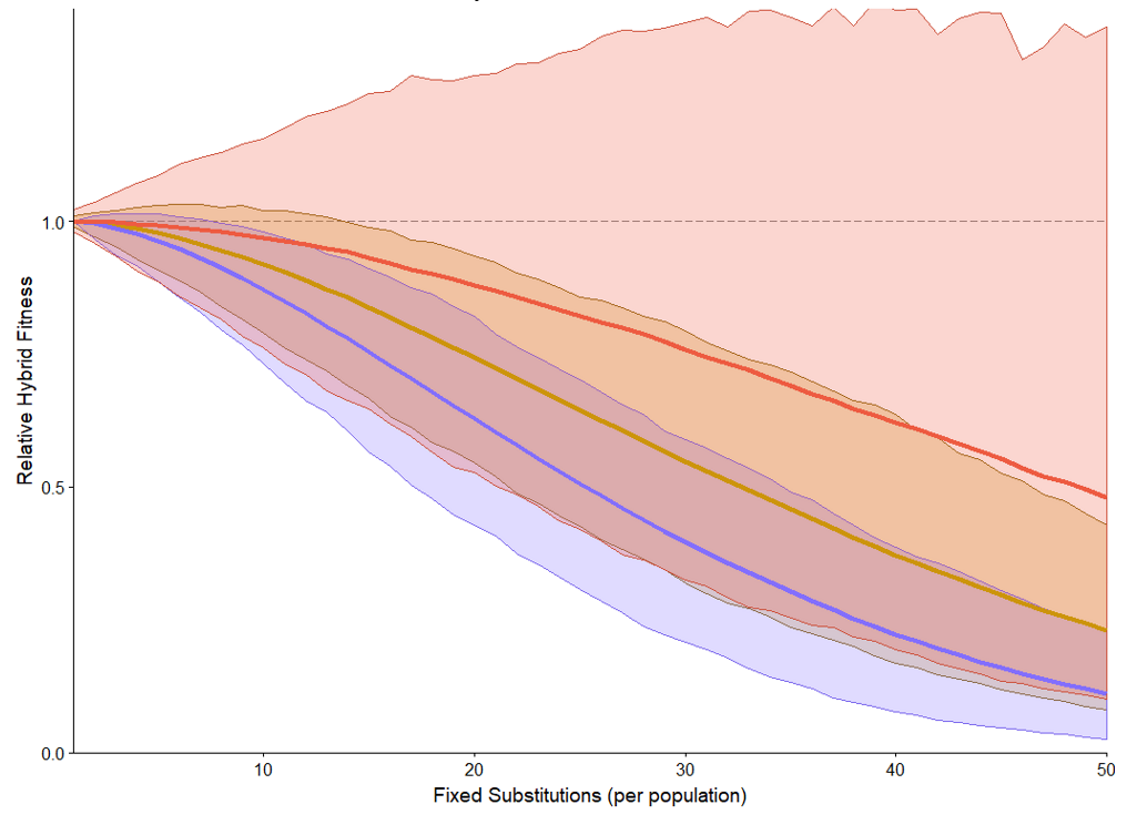

Supplement: S12 Fig — Relative hybrid fitness when assumptions about dominance of epistasis are modified. We consider our simulations in which α2 = 0.5. Since hybrids will only experience epistasis of strength α1ε, we reanalyze their fitness assuming epistasis seen in hybrids is recessive (blue, α1 = 0), additive (yellow, α1 = 1/2 α2) and dominant (red, α1 = α2). Solid lines represent means of simulations, shaded areas are 95% confidence intervals. As suggested by our model, highly dominant interactions lead to slower decline in fitness. Simulations demonstrate that a secondary effect is increased variance in observed hybrid fitness. (TIF) [file pgen.1008125.s013.tif]

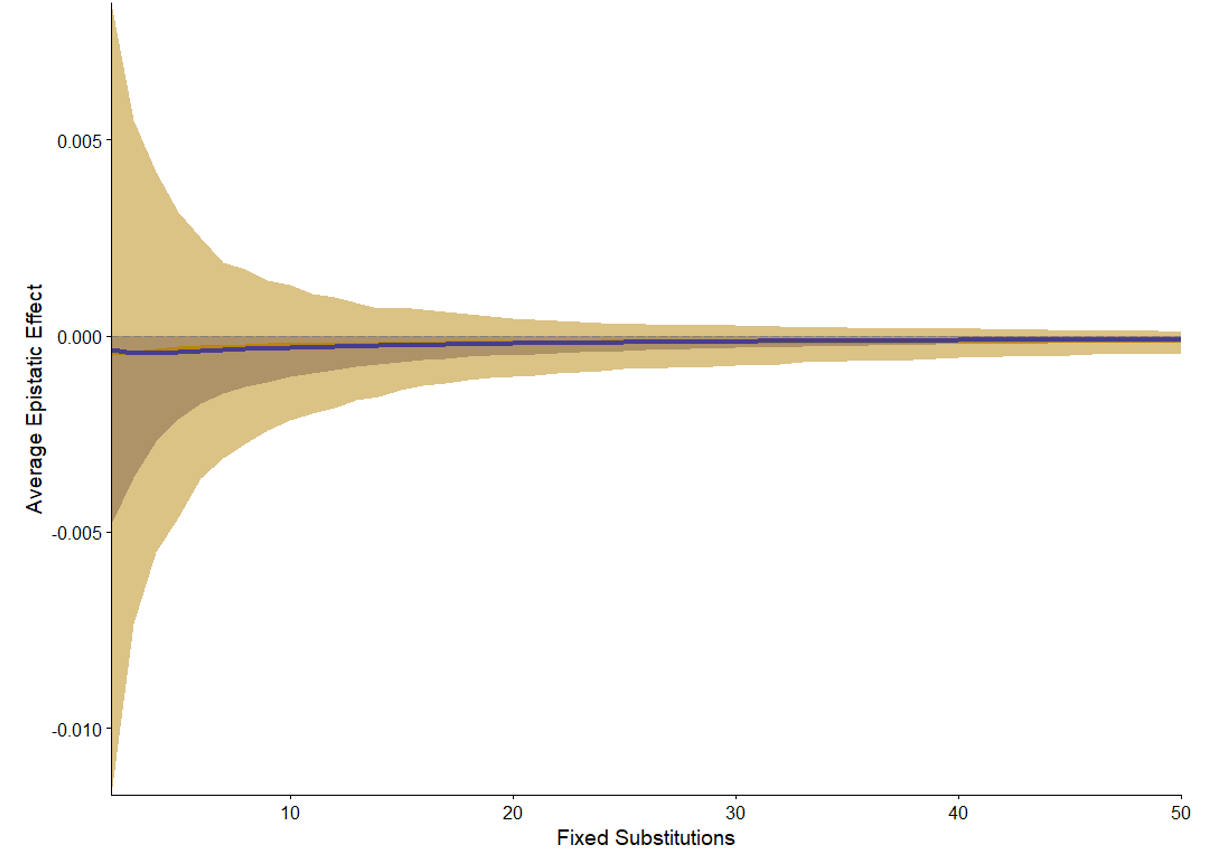

Supplement: S13 Fig — Average epistatic effects within (purple) and between (tan) populations. While variance of within population effects is lower than that of between populations, the two rapidly converge to the same slightly negative value. Shaded areas represent the 95% confidence interval of 2000 simulations, solid lines are means. (TIF) [file pgen.1008125.s014.tif]

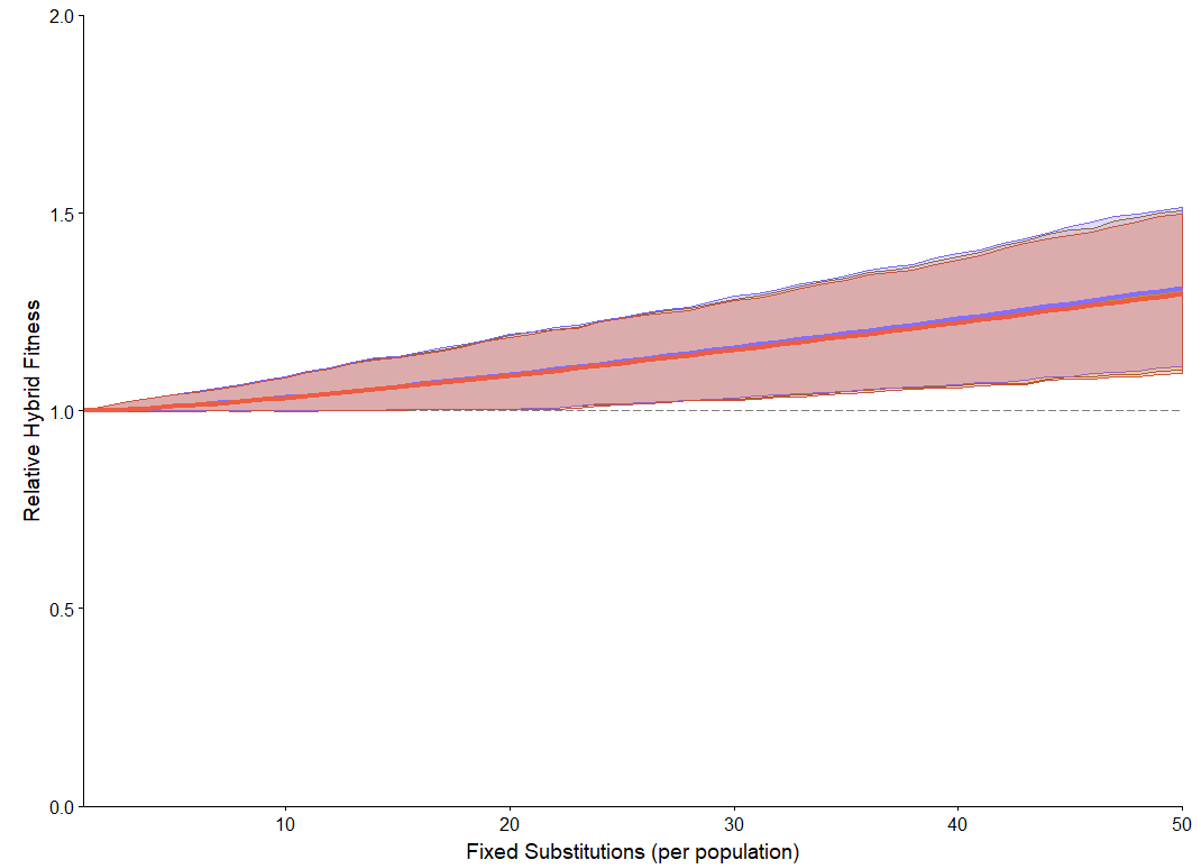

Supplement: S14 Fig — Relative hybrid fitness in simulations when epistatic effects are nearly completely recessive as mutations are fixed (α2 = 0.01). Hybrid fitness depends very little on whether epistasis seen in hybrids is recessive (blue, α1 = 0), additive (yellow, α1 = 1/2 α2), or dominant (red, α1 = α2). Hybrid fitness therefore increases linearly with respect to fixed substitutions, proportional to the average epistatic effect. (TIF) [file pgen.1008125.s015.tif]

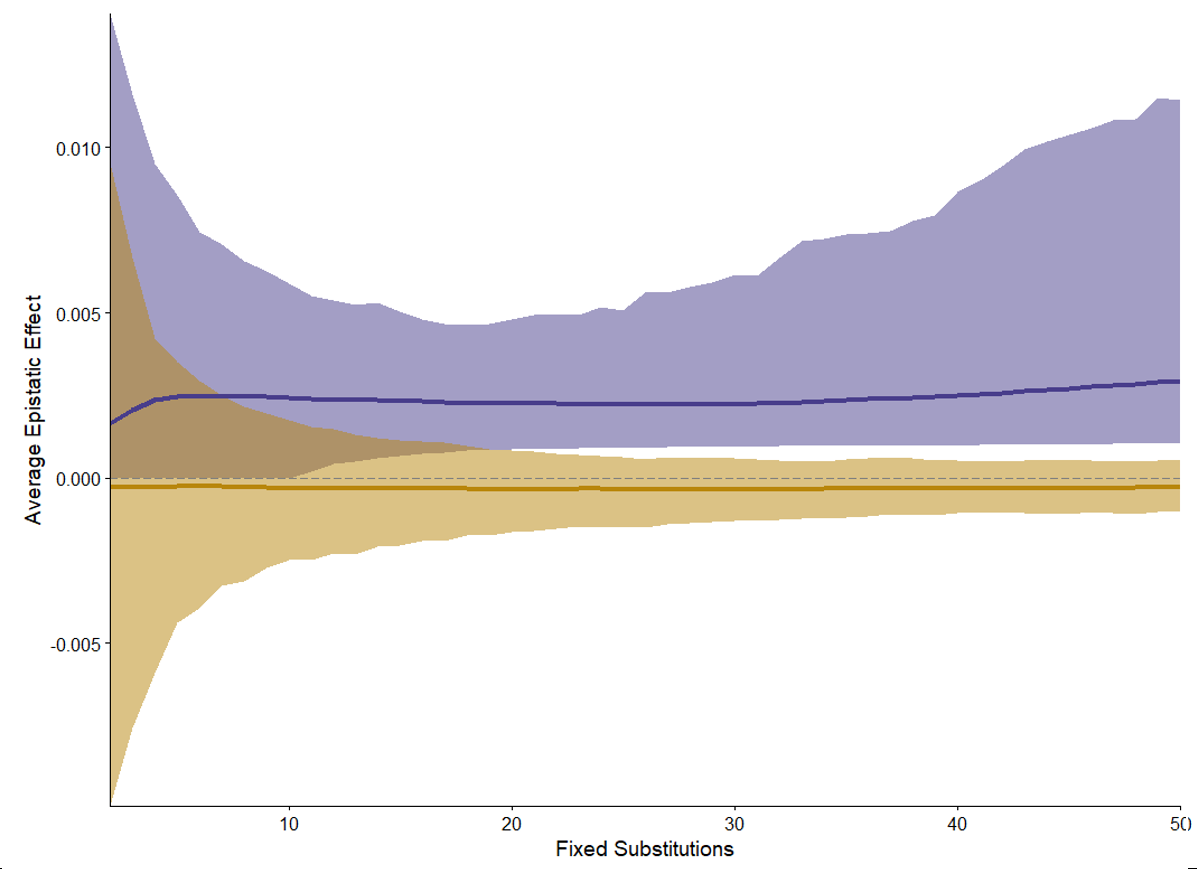

Supplement: S15 Fig — The average epistatic effect found among mutations fixed in the same population (purple) or in different populations (tan), when epistatic effects are largely dominant. Few deleterious epistatic effects can be fixed within the same population, and so epistasis within rises rapidly and continues to increase as populations diverge. Between population epistasis behaves the same as in simulations where epistasis additively dominant. Shaded areas represent the 95% confidence interval of 2000 simulations, solid lines are means. (TIF) [file pgen.1008125.s016.tif]

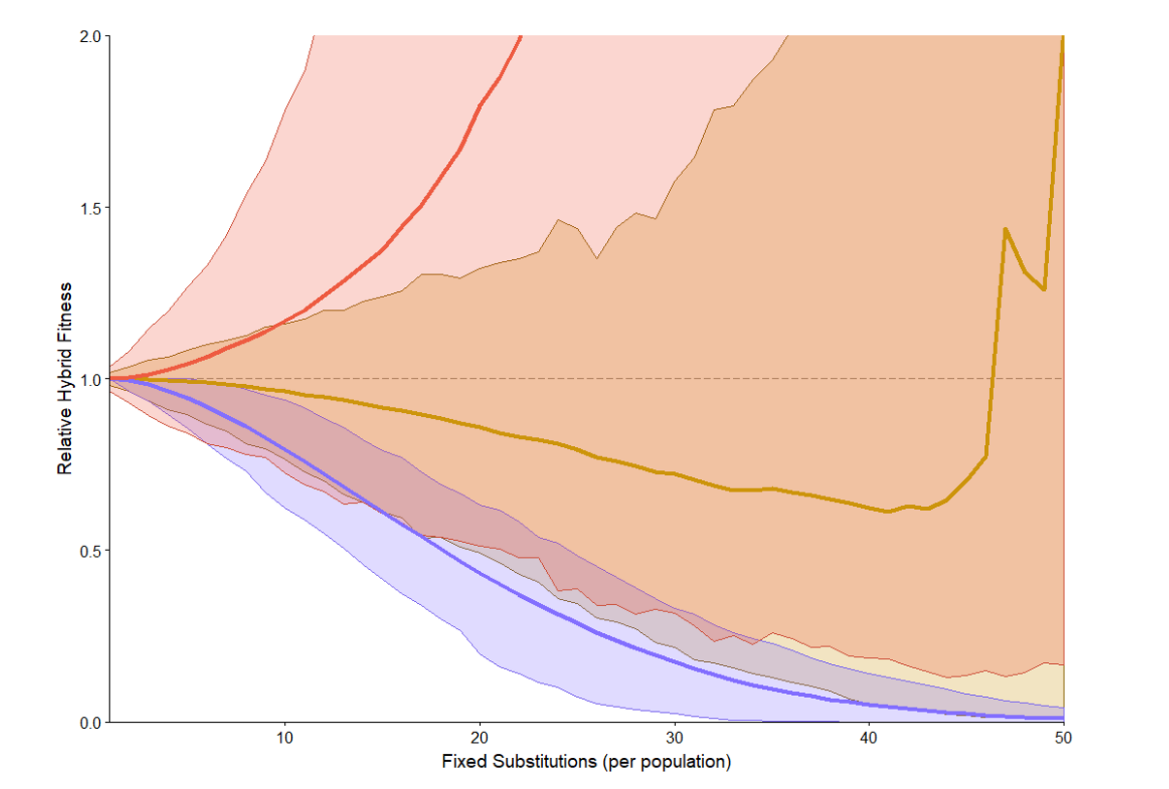

Supplement: S16 Fig — Relative hybrid fitness in simulations when epistatic effects are nearly completely dominant as they are fixed (α2 = 0.95). Because within population epistasis is large, hybrid fitness depends heavily on whether epistasis seen in hybrids is recessive (blue, α1 = 0), additive (yellow, α1 = 1/2 α2), or dominant (red, α1 = α2). When effects are recessive, hybrids lose out on co-adapted blocks and rapidly lose fitness, while interactions that display nearly the same strength as seen in parents (red) lead to extreme heterosis due to combinations of positive epistatic effects from both parents. (TIF) [file pgen.1008125.s017.tif]
